# Practical synthesis of aryl-2-methyl-3-butyn-2-ols from aryl bromides via conventional and decarboxylative copper-free Sonogashira coupling reactions

**DOI:** 10.3762/bjoc.10.36

**Published:** 2014-02-12

**Authors:** Andrea Caporale, Stefano Tartaggia, Andrea Castellin, Ottorino De Lucchi

**Affiliations:** 1 Dipartimento di Scienze Molecolari e Nanosistemi – Università Ca' Foscari Venezia, Dorsoduro 2137, I-30123 Venice, Italy; 2 F.I.S. – Fabbrica Italiana Sintetici S.p.A., Alte di Montecchio Maggiore (Vicenza), Viale Milano 26, I-36075 Vicenza, Italy

**Keywords:** alkynes, decarboxylative couplings, Erlotinib, palladium, propiolic acid

## Abstract

Two efficient protocols for the palladium-catalyzed synthesis of aryl-2-methyl-3-butyn-2-ols from aryl bromides in the absence of copper were developed. A simple catalytic system consisting of Pd(OAc)_2_ and P(*p*-tol)_3_ using DBU as the base and THF as the solvent was found to be highly effective for the coupling reaction of 2-methyl-3-butyn-2-ol (**4**) with a wide range of aryl bromides in good to excellent yields. Analogously, the synthesis of aryl-2-methyl-3-butyn-2-ols was performed also through the decarboxylative coupling reaction of 4-hydroxy-4-methyl-2-pentynoic acid with aryl bromides, using a catalyst containing Pd(OAc)_2_ in combination with SPhos or XPhos in the presence of tetra-*n*-butylammonium fluoride (TBAF) as the base and THF as the solvent. Therefore, new efficient approaches to the synthesis of terminal acetylenes from widely available aryl bromides rather than expensive iodides and using **4** or propiolic acid rather than TMS-acetylene as inexpensive alkyne sources are described.

## Introduction

The Sonogashira coupling reaction of aryl or alkenyl halides with terminal acetylenes is the most straightforward method for the preparation of substituted alkynes [1–6], which are extensively used as building blocks in a great number of applications including the synthesis of pharmaceuticals [7–9], natural products and organic functional materials [10–13].

The Sonogashira coupling reaction is usually carried out under Pd/Cu catalysis, in which the palladium has the function to promote the cross-coupling of an aryl fragment, added via oxidative addition of the corresponding halide, with an alkynyl residue to provide a disubstituted acetylene after reductive elimination. The activated acetylide species for the coupling process is generated from the reaction of a terminal alkyne with copper in the presence of a base and is transferred on the palladium site via transmetallation.

In order to improve the efficacy of the reaction, several variants have been introduced to perform efficiently this reaction with regard to synthetic and industrial aspects, including protocols in aqueous media [14–17], amine-free [18] or solvent-free conditions [19]. Moreover efficient reusable Pd catalysts [20–24] and even palladium-free methodologies [25–27] were developed. Copper-free reaction protocols [28–41] are highly promising for industrial applications as the presence of copper could lead to the formation of diyne byproducts, causing an irreversible loss of precious material, and complicates the recycling of the palladium catalyst since the two metals are difficult to separate.

Sonogashira reactions can be used for the syntheses of terminal alkynes from aryl halides through the coupling with an alkyne source such as trimethylsilylacetylene (TMSA) or 2-methyl-3-butyn-2-ol (**4**) in presence of a Cu/Pd bimetallic catalyst, followed by the basic cleavage of the protecting group [42–46]. Terminal alkynes are often used as starting materials for the synthesis of disubstituted acetylenes through a second coupling process with another aryl halide.

Decarboxylative couplings have also been reported as an efficient synthetic tool for the synthesis of disubstituted alkynes. In fact, the use of propiolic acid as an alkyne source has opened new possibilities in the synthesis of acetylenic compounds, since many examples leading to diaryl- (sp–sp^2^ coupling), benzyl- (sp–sp^3^ coupling) and diaryl diacetylenes (sp–sp coupling) in addition to examples of carbon-heteroatom couplings have been reported in the recent literature [47–65]. The convenience in using propiolic acid as the alkyne source in the synthesis of symmetrical and unsymmetrical diarylacetylenes resides not only in its low cost, but also in the possibility to carry out a conventional and a decarboxylative Sonogashira coupling reaction in sequence at the two sp carbons with two different aryl halides. Therefore a disubstituted alkyne can be obtained in a one-pot protocol with a considerable reduction of synthetic and purification steps with respect to traditional procedures [51–52,62]. Instead, when looking to the preparation of terminal alkynes from propiolic acid derivatives, the Cu-catalyzed protodecarboxylation of alkynoic acids [67–68] or the Pd-catalyzed decarboxylation of allyl propiolates [69] have been reported, but these methods are limited to a narrow class of substrates and are not well suited for the preparation of aryl alkynes. More recently an optimized protocol for the synthesis of terminal arylalkynes from propiolic acid has been reported by Lee, in which the Pd-catalyzed coupling of propiolic acid with an aryl iodide is followed by the addition of the Cu-catalyst, which promotes the protodecarboxylation of the arylpropiolic acid intermediate to the desired alkyne in good yields [70].

**Scheme 1:**
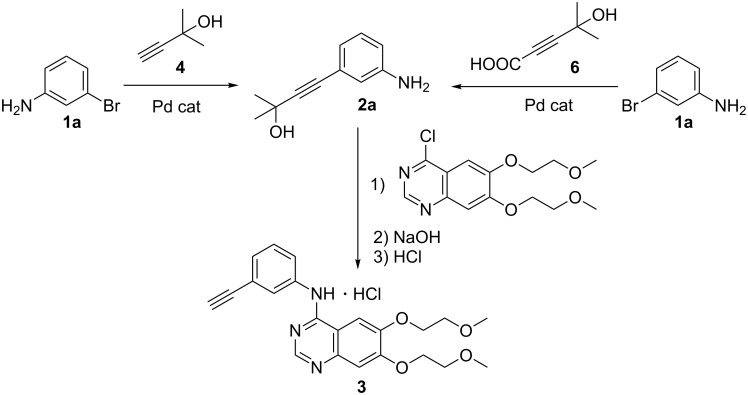
Conventional (from the left) and decarboxylative (from the right) Pd-catalyzed Sonogashira coupling reactions for the preparation of 4-(3-aminophenyl)-2-methyl-3-butin-2-ol (**2a**), which is used as an intermediate for the synthesis of Erlotinib hydrochloride (**3**).

Acetylenic compounds and Sonogashira coupling reactions have found many applications in the synthesis of pharmaceutical substances. During the investigation about a possible alternative preparation of 2-methyl-4-(3-aminophenyl)-3-butyn-2-ol (**2a**) to improve a key-step in the synthesis of Erlotinib, an important anticancer pharmaceutical intermediate [71] and a promising anti-Alzheimer [72], we found that the Pd/Cu-catalyzed Sonogashira coupling reaction of 3-bromoaniline (**1a**) with **4** is the actual industrial process for the synthesis of **2a** in high yield [73–75] (Scheme 1).

However, when the reaction is performed at industrial scale the presence of copper creates several problems in particular during the purification step. Moreover, the use of copper in Sonogashira reactions should be avoidable because of unwanted side homo-couplings of the acetylenic substrate, causing an irreversible loss of precious starting material. Therefore, the Pd-catalyzed coupling reaction of **4** or a propiolic acid derivative with 3-bromoaniline, in order to approach the synthesis of 3-aminophenylacetylene without the use of copper, would be highly desiderable [76].

Although many examples of copper-free Sonogashira reactions between aryl halides and terminal alkynes for the synthesis of disubstituted acetylenes have been reported in the literature [9], few specific protocols for **4** were presented [77–79]. Successful copper free synthesis of 4-aryl-2-methyl-3-butyn-2-ols from aryl bromides have been performed by carrying out the reaction in piperidine as the solvent [39,78] or by using aminophosphines [18] as well as phenanthryl imidazolium carbenes as the catalyst ligands [79]. A more practical methodology has been reported by Shirakawa in which a Pd(OAc)_2_/PPh_3_ catalyst system in DMSO and in the presence of K_3_PO_4_ as the base was able to couple aryl bromides with terminal alkynes, including a couple of examples with 2-methyl-3-butyn-2-ol, in moderate yield [35]. Two simple reaction protocols for the copper-free coupling of **4** have been also reported for iodo nitroxides [30] and cyclopropyl iodides [29].

Herein, we present two practical and very efficient synthetic methods for the preparation of aryl-2-methyl-3-butyn-2-ols from aryl bromides via Pd-catalyzed conventional and decarboxylative coupling reactions by using **4** or 4-hydroxy-4-methyl-2-pentynoic acid as the alkyne sources and without the use of copper. These methods have been optimized for the synthesis of 2-methyl-4-(3-aminophenyl)-3-butyn-2-ol, which has never been prepared through a Cu-free coupling process and which is also a pharmaceutical intermediate for Erlotinib.

The present work provides also a more detailed description about the coupling process and a more extended rection scope that was not included in a previous patent application by our group, in which the decarboxylative coupling reaction for the synthesis of intermediate **2a** has been firstly described [76].

## Results and Discussion

In order to perform efficiently the palladium-catalyzed coupling reaction between 3-bromoaniline (**1a**) and **4** to give 2-methyl-4-(3-aminophenyl)-3-butyn-2-ol (**2a**) (Table 1), we screened different bases, solvents and ligands in the absence of copper. We firstly examined the coupling reaction using a catalytic system based on palladium acetate and triphenylphosphine in presence of TBAF (tetrabutylammonium fluoride) as the base. After using different solvents, we found that THF provided the best results with regard to toluene and more polar media such as DMF, affording the product in 61% yield (Table 1, entries 1–3). The use of inorganic bases as well as common organic amines, typically employed in conventional Pd/Cu Sonogashira coupling protocols, resulted in poor yields of our desired product (Table 1, entries 4–6). Piperidine, which has been reported to efficiently perform copper-free coupling reactions of **4** with polycyclic aryl bromides [77], gave only 17% yield with our substrate (Table 1, entry 6). Instead, a decisive increase of the yield was observed by replacing TBAF with DBU, which furnished product **2a** in 84% yield (Table 1, entry 7). A further improvement was found by modifying the catalyst with different ligands (Table 1, entries 7–11). In fact, changing of PPh_3_ with P(*p*-tol)_3_ allowed to obtain **2a** in 89% yield (Table 1, entry 9), whereas other monodentate or chelating phosphines displayed a lower efficiency. Pd sources other than Pd(OAc)_2_ were also used but did not show positive effects.

**Table 1:** Synthesis of 2-methyl-4-(3-aminophenyl)-3-butyn-2-ol-from 3-bomoaniline and 2-methyl-3-butyn-2-ol.^a^

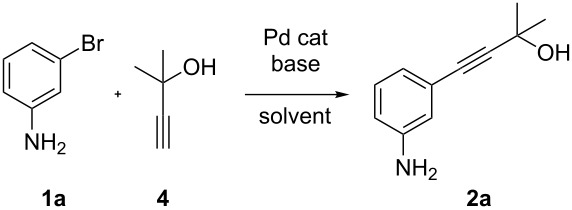				

Entry	Solvent	Base	Ligand	Yield (%)

1	toluene	TBAF	PPh_3_	32
2	THF	TBAF	PPh_3_	61
3	DMF	TBAF	PPh_3_	9
4	THF	K_2_CO_3_	PPh_3_	14
5	THF	NEt_3_	PPh_3_	5
6	THF	piperidine	PPh_3_	17
7	THF	DBU	PPh_3_	84
8	THF	DBU	P(*p*-FC_6_H_4_)_3_	75
9	THF	DBU	P(*p*-tol)_3_	89
10	THF	DBU	P(*o*-tol)_3_	45
11	THF	DBU	dppe	50

^a^Reaction conditions: **1a** (1.0 mmol), **4** (1.2 mmol), Pd(OAc)_2_ (0.03 mmol), ligand (6 mol %), base (3 equiv), 6 h, 80 °C. Yields were determined by GC using tetradecane as an internal standard.

Having thus found an optimized method for the cooper free palladium-catalyzed coupling of 3-bromoaniline (**1a**) with 2-methylbut-3-yn-2-ol (**4**), we next examined wherever this protocol could be applied to a more general class of substrates.

As stated in Table 2, good to excellent yields were obtained by using different aryl bromides including electron-rich, electron-poor, sterically crowded and heterocyclic derivatives. Moreover, the coupling reaction seems to be very efficient and selective with regard to different functional groups placed on the aryl bromide, including acetamido, dimethylamino, methoxy, chloro, fluoro, trifluoromethyl, nitro, methoxycarbonyl and cyano groups. Bromonaphthalene (**1j**) and 3-bromopyridine (**1n**) reacted smoothly.

**Table 2:** Copper-free palladium-catalyzed coupling of 2-methylbut-3-yn-2-ol (**4**) with aryl bromides.^a^

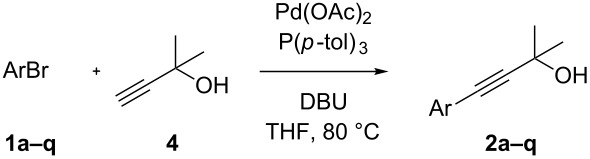							

Entry	ArBr **1**	Product **2**	Yield (%)	Entry	ArBr **1**	Product **2**	Yield (%)

1	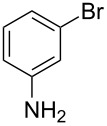 **1a**	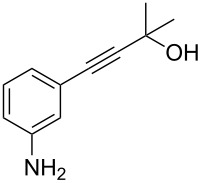 **2a**	86	10	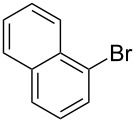 **1j**	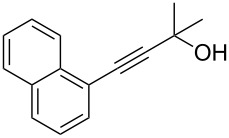 **2j**	95
2	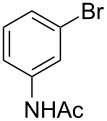 **1b**	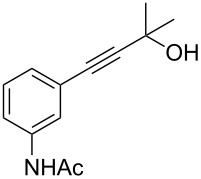 **2b**	95	11	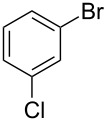 **1k**	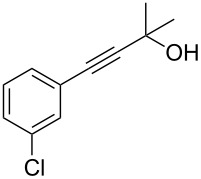 **2k**	96
3	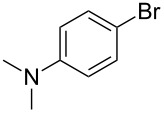 **1c**	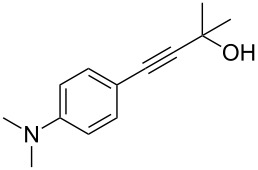 **2c**	73	12	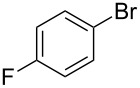 **1l**	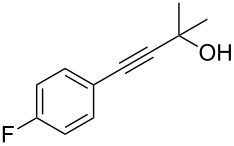 **2l**	96
4	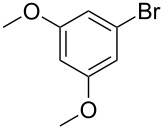 **1d**	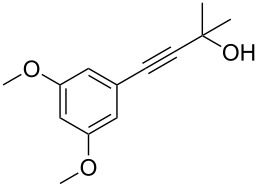 **2d**	89	13	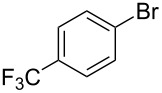 **1m**	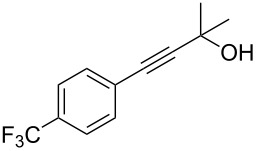 **2m**	89
5	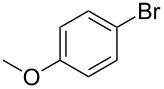 **1e**	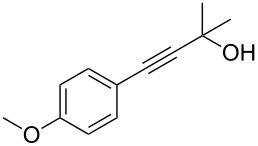 **2e**	70	14	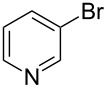 **1n**	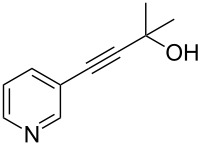 **2n**	84
6	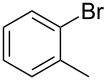 **1f**	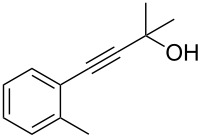 **2f**	80	15	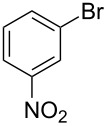 **1o**	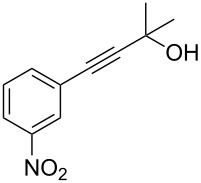 **2o**	77
7	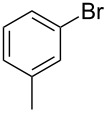 **1g**	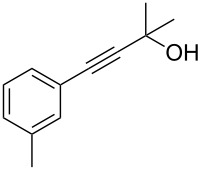 **2g**	89	16	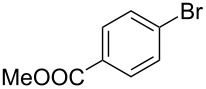 **1p**	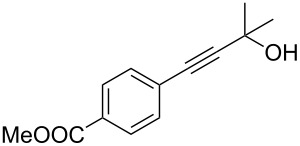 **2p**	72
8	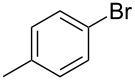 **1h**	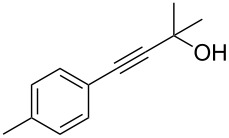 **2h**	87	17	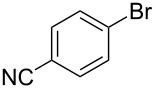 **1q**	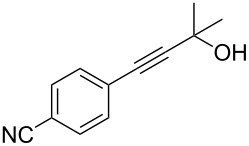 **2q**	92
9	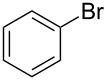 **1i**	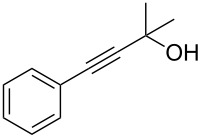 **2i**	94				

^a^Reaction conditions: **1a**–**q** (1.0 mmol), 2-methylbut-3-yn-2-ol (**4**) (1.2 mmol), Pd(OAc)_2_ (3 mol %), P(*p*-tol)_3_ (6 mol %), DBU (3 mmol), 80 °C, 6 h. Yields refer to isolated products.

Bromobenzenes bearing a methyl groups in the *ortho*, *meta* and *para* positions gave the corresponding products in similar good yields. Thus, the reaction can be applied to a wide substrate range witnessing the attractiveness and the generality of this protocol. It is also worth noting that these conditions are also highly practical and well suited to scale-up the reaction, since inexpensive **4** can be efficiently coupled with aryl bromides instead of iodides by using common solvents and ligands for the palladium catalyst.

**Scheme 2:**
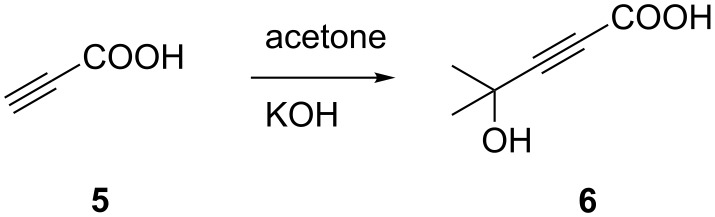
Protection of propiolic acid with acetone.

The copper-free synthesis of 4-aryl-2-methyl-3-butyn-2-ols was also approached through a decarboxylative coupling reaction in which a derivative of propiolic acid (**5**) was used as the alkyne source. In order to prevent the possible double coupling process due to the high reactivity of the terminal C(sp)-H, the protection of the acetylenic proton of **5** was necessary. Analogously to acetylene [80–81], the protection of propiolic acid with acetone was carried out in presence of an excess of KOH (6.0 equiv) at room temperature (Scheme 2).

The screening of the optimal palladium catalyst for the Pd-catalyzed coupling reaction of 3-bromoaniline with 4-hydroxy-4-methyl-2-pentynoic acid (**6**) was studied by using different bases, ligands and Pd sources (Table 3). In accordance with the reported data, tetrabutylammonium fluoride (TBAF) [64,66] was found to be able to promote the decarboxylative coupling at 80 °C (Table 3, entry 5) whereas Cs_2_CO_3_ [63], CsF or organic amines were less effective in promoting this reaction (Table 3, entries 1–4).

**Table 3:** Palladium-catalyzed coupling reaction of 4-hydroxy-4-methyl-2-pentynoic acid and *m*-bromoaniline.^a^

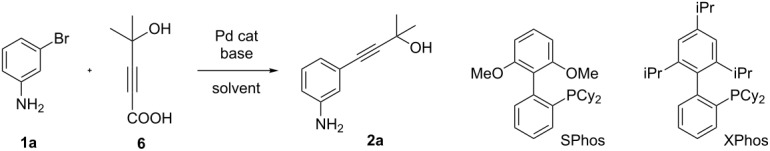					

Entry	Solvent	Base	Pd	Ligand	Yield (%)

1	THF	Cs_2_CO_3_	Pd(OAc)_2_	SPhos	5
2	THF	CsF	Pd(OAc)_2_	SPhos	7
3	THF	NH(iPr)_2_	Pd(OAc)_2_	SPhos	4
4	THF	DBU	Pd(OAc)_2_	SPhos	42
5	THF	TBAF	Pd(OAc)_2_	SPhos	80
6	NMP	TBAF	Pd(OAc)_2_	SPhos	76
7	THF	TBAF	Pd(OAc)_2_	XPhos	55
8	THF	TBAF	Pd_2_(dba)_3_	SPhos	89
9	THF	TBAF	(PdallylCl)_2_	SPhos	79

^a^Reaction conditions: **1a** (0.5 mmol), **6** (0.63 mmol), Pd source (5 mol %), ligand (7.5 mol %), base (3 equiv), 14 h, 80 °C. Yields were determined by GC using tetradecane as an internal standard.

The use of bulky electron-rich biarylphosphines as ligands for the palladium catalyst seems also to play a crucial role in promoting the coupling reaction. In fact, SPhos (Table 3, entry 5) resulted the best ligand with respect to XPhos (Table 3, entry 7), whereas other simple monodentate phosphines, such as P*t-*Bu_3_ or PPh_3_, seem not to be able to promote efficiently the reaction (data not shown). The change of the Pd source to Pd_2_(dba)_3_ led to a further increase of the yield up to 89% (Table 3, entry 8), while other precursors decreased the performances of the system. Concerning the solvent media, similar yields were obtained using THF or NMP (Table 3, entries 5 and 6), but when toluene or dioxane were used, the yields were not satisfactory.

With an optimized system for the coupling of **6** with *m*-bromoaniline in hand, we next investigated the scope of the reaction (Table 4).

**Table 4:** Palladium catalyzed coupling of 4-hydroxy-4-methyl-2-pentynoic acid (**6**) with aryl bromides.^a^

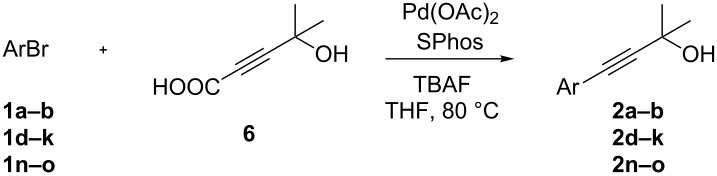							

Entry	ArBr **1**	Product **2**	Yield (%)	Entry	ArBr **1**	Product **2**	Yield (%)

1	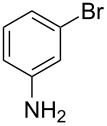 **1a**	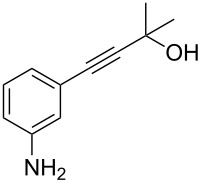 **2a**	79 (86)^b^	7	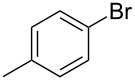 **1h**	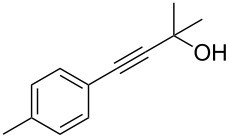 **2h**	72
2	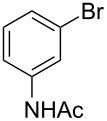 **1b**	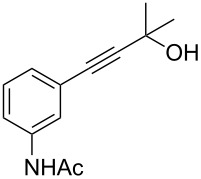 **2b**	80	8^c^	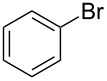 **1i**	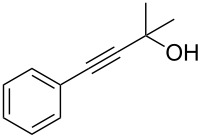 **2i**	68
3	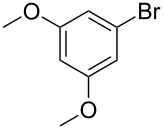 **1d**	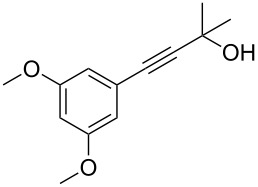 **2d**	69	9	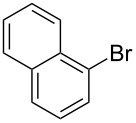 **1j**	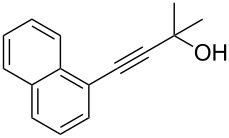 **2j**	53
4	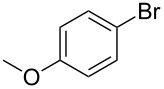 **1e**	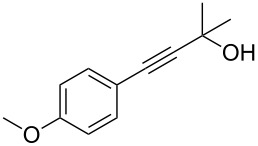 **2e**	56	10^c^	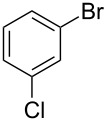 **1k**	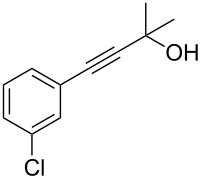 **2k**	25
5	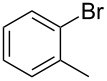 **1f**	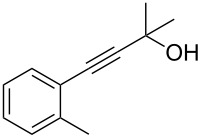 **2f**	78	11^c^	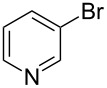 **1n**	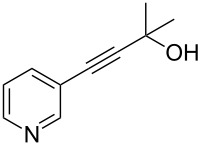 **2n**	61
6	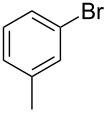 **1g**	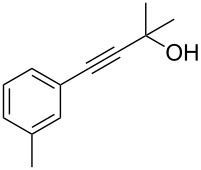 **2g**	82	12^c^	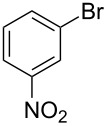 **1o**	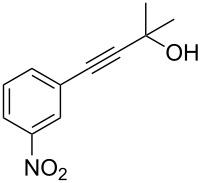 **2o**	80

^a^Reaction conditions: **1** (0.5 mmol), 4-hydroxy-4-methyl-2-pentynoic acid (**6**) (0.63 mmol), Pd(OAc)_2_ (5 mol %), SPhos (7.5 mol %), TBAF·3H_2_O (1.5 mmol), 80 °C, 14 h. Yields refer to isolated products. ^b^Pd_2_(dba)_3_ was used instead of Pd(OAc)_2_. ^c^4-Hydroxy-4-methyl-2-pentynoic acid (1 mmol) and XPhos instead of SPhos were used.

A variety of aryl bromides bearing electron-rich, electron-neutral and electron-poor substituents were coupled with 4-hydroxy-4-methyl-2-pentynoic acid (**6**) affording the corresponding products in good yields. As can be seen from the examples listed in Table 4 several functional groups were tolerated including acetamido, methoxy and nitro groups. The reaction has been applied also to heterocyclic and polycyclic aryl bromides.

A little modification with respect to 3-bromoaniline (**1a**) was necessary in order to successfully apply the protocol to all substrates used for the synthesis of 4-aryl-2-methyl-3-butyn-2-ols through the decarboxylative coupling approach. In fact, electron rich aryl bromides reacted smoothly with **6** when using a system containing Pd(OAc)_2_/SPhos as the catalyst [82], TBAF as the base in THF as solvent. The corresponding alkynes were therefore obtained in good yields (60–80% range), especially for those substrates bearing methyl, methoxy and amino groups (Table 4, entries 1–8). However, in the case of bromobenzene and aryl bromides bearing electron withdrawing groups, the corresponding products were obtained with satisfactory yields only when using 2 equiv of **4** with respect to the substrate and replacing SPhos with XPhos as ligand for the palladium catalyst (Table 4, entries 9–12). In these cases we observed also the formation of the symmetrical diarylacetylene as byproduct derived from the starting aryl bromides in a 2–5% yield range. Likely, the acetone protecting group is cleaved from protected terminal alkyne even under such mild conditions and therefore, the terminal alkyne generated in situ couples with another molecule of the starting aryl bromide to provide the symmetrical acetylene.

Finally, it is worth noting that the preparation of product **2a** has been also reported through the acid hydrolysis of the acetamido group of **2b** or by the reduction of the nitro group of **2o** [76], however the direct coupling of 3-bromoaniline with **4** or **6** clearly remains the most efficient approach to the synthesis of 3-ethynylaniline.

## Conclusion

Two simple and efficient protocols for the preparation of aryl-2-methyl-3-butyn-2-ols from aryl bromides, which are inexpensive intermediates for the preparation of terminal alkynes, have been described. A copper-free, palladium catalyst generated from Pd(OAc)_2_ and P(*p-*tol)_3_ allowed the coupling of aryl bromides with **4** in THF. The use of DBU as the base seems to play a crucial role to efficiently perform this coupling reaction on a wide scope of substrates without the use of copper. Moreover, the palladium-catalyzed decarboxylative coupling reaction of 4-hydroxy-4-methyl-2-pentynoic acid with aryl bromides for the preparation of aryl-2-methyl-3-butyn-2-ols has been investigated. The decarboxylative coupling was carried out by using a catalyst generated in situ from Pd(OAc)_2_ and a bulky electron-rich phospine, such as SPhos or XPhos, in THF as the solvent and in presence of TBAF as the base. By approaching the synthesis of aryl-2-methyl-3-butyn-2-ols through decarboxylative coupling reactions of 4-hydroxy-4-methyl-2-pentynoic acid, we found a good compatibility with differently substituted aryl bromides.

## Experimental

All reactions, if not stated otherwise, were performed in oven-dried glassware under an argon atmosphere containing a teflon-coated stirring bar and a dry septum. Chemicals and solvents were either purchased (puriss. p.a.) from commercial suppliers or purified by standard techniques. All reactions were monitored by GC using tetradecane as an internal standard. Response factors of the products with regard to tetradecane were obtained experimentally by analyzing known quantities of the substances. GC analyses were carried out using an HP-5 capillary column (phenyl methyl siloxane, 30 m × 320 × 0.25, 100/2.3-30-300/3, 2 min at 50 °C, heating rate 25 °C/min, 3 min at 250 °C). Column chromatography was performed with 230–400 mesh silica-gel. NMR spectra were obtained on a Bruker AVANCE 300 spectrometer (300 MHz) using CDCl_3_ as solvent, 300 MHz and 75 MHz, respectively. Mass spectral data were acquired on a Trace GC–MS 2000 ThermoQuest. Melting points were measured on a Büchi 535.

### General procedure for the preparation of arylalkynes **2a–q** (method A)

An oven-dried 20 mL Schlenk tube equipped with a magnetic stirring bar and a rubber septum was charged with Pd(OAc)_2_ (6.7 mg, 30 µmol) and tri(*p-*tolyl)phosphine (18.2 mg, 60 µmol). After purging the vessel with alternating vacuum and nitrogen cycles, degassed THF (3 mL), 1,8-diazabicycloundec-7-ene (450 µL, 3.0 mmol), 2-methylbut-3-yn-2-ol (120 µL, 1.24 mmol) and aryl bromides **1a–q** (1 mmol) were added via syringe (solid aryl bromides were added as solution in degassed THF) and the mixture was stirred at 80 °C for 6 h. After cooling to rt the mixture was diluted with water (20 mL) and extracted with AcOEt (3 × 20 mL). The combined organic extracts were washed with H_2_O (20 mL), saturated aqueous NaCl (20 mL), dried over MgSO_4_ and concentrated in vacuum. The crude product was purified by silica gel chromatography (eluant hexane/Et_2_O gradient) affording the corresponding products **2a–q**.

### General procedure for the preparation of arylalkynes **2a,b**, **2d–h** and **2j** (method B)

An oven-dried 20 mL Schlenk tube equipped with a magnetic stirring bar and a rubber septum was charged with Pd(OAc)_2_ (5.6 mg, 25 µmol), 2-dicyclohexylphosphino-2',6'-dimethoxybiphenyl (15.4 mg, 37.5 µmol) and 4-hydroxy-4-methyl-2-pentynoic acid (**6**, 80 mg, 0.63 mmol). After purging the vessel with alternating vacuum and nitrogen cycles, a degassed solution of TBAF∙3H_2_O (0.47 g, 1.5 mmol) in THF (3 mL) was added. Aryl bromides **1a**,**b**, **1d–h** and **1j** (0.5 mmol) were added via syringe (solid aryl bromides were added as solution in degassed THF) and the mixture was stirred at 80 °C for 14 h. After cooling to rt the mixture was diluted with water (20 mL) and extracted with AcOEt (3 × 20 mL). The combined organic extracts were washed with H_2_O (20 mL), saturated aqueous NaCl (20 mL), dried over MgSO_4_ and concentrated in vacuum. The crude product was purified by silica gel chromatography (eluant cyclohexane/Et_2_O or EtOAc gradient) affording the corresponding products **2a**,**b**, **2d–h** and **2j**.

### General procedure for the preparation of arylalkynes **2i**, **2k**, **2n** and **2o** (method C)

An oven-dried 20 mL Schlenk tube equipped with a magnetic stirring bar and a rubber septum was charged with Pd(OAc)_2_ (5.6 mg, 25 µmol), 2-Dicyclohexylphosphino-2′,4′,6′-triisopropylbiphenyl (17.9 mg, 37.5 µmol), and 4-hydroxy-4-methyl-2-pentynoic acid (**6**, 128 mg, 1.00 mmol). After purging the vessel with alternating vacuum and nitrogen cycles, a degassed solution of TBAF∙3H_2_O (0.47 g, 1.5 mmol) in THF (3 mL) was added. Aryl bromides **1i**, **1k**, **1n** and **1o** (0.5 mmol) were added via a syringe (solid aryl bromides were added as solution in degassed THF) and the mixture was stirred at 80 °C for 14 h. After cooling to rt the mixture was diluted with water (20 mL) and extracted with AcOEt (3 × 20 mL). Combined organic extracts were washed with H_2_O (20 mL), saturated aqueous NaCl (20 mL), dried over MgSO_4_ and concentrated in vacuum. The crude product was purified by silica gel chromatography (eluant cyclohexane/Et_2_O or EtOAc gradient) affording the corresponding products **2i**, **2k**, **2n** and **2o**.

## Supporting Information

File 1 Description of all procedures and characterization data of all new compounds.
