# Supplementary material for: Practical synthesis of aryl-2-methyl-3-butyn-2-ols from aryl bromides via conventional and decarboxylative copper-free Sonogashira coupling reactions
Source: Beilstein J Org Chem. 2014 Feb 12;10:384–93. doi: 10.3762/bjoc.10.36 (PMC3943628; doi:10.3762/bjoc.10.36)

Supporting Information

for

Practical synthesis of aryl-2-methyl-3-butyn-2-ols from aryl bromides via conventional and decarboxylative copper-free Sonogashira coupling reactions

Andrea Caporale *[1], Stefano Tartaggia [1], Andrea Castellin[2] and Ottorino De Lucchi[1]

Address: [1]Dipartimento di Scienze Molecolari e Nanosistemi – Università Ca' Foscari Venezia, Dorsoduro 2137, I-30123 Venice, Italy and [2]F.I.S. – Fabbrica Italiana Sintetici S.p.A., Alte di Montecchio Maggiore (Vicenza), Viale Milano 26, I-36075 Vicenza, Italy

Email: Andrea Caporale - caporaleandrea74@gmail.com

*Corresponding author

Description of all procedures and characterization data of all new compounds

General. All reactions, if not stated otherwise, were performed in oven-dried glassware under an argon atmosphere containing a teflon-coated stirring bar and dry septum. Chemicals and solvents were either purchased (puriss. p.A.) from commercial suppliers or purified by standard techniques. All reactions were monitored by GC using tetradecane as an internal standard. Response factors of the products with regard to tetradecane were obtained experimentally by analyzing known quantities of the substances. GC analyses were carried out using an HP-5 capillary column (phenyl methyl siloxane, 30 m × 320 × 0.25, 100/2.3-30-300/3, 2 min at 50 °C, heating rate 25 °C/min, 3 min at 250 °C). Column chromatography was performed with 230–400 mesh silica-gel. NMR spectra were obtained on a Bruker AVANCE 300 spectrometer (300 MHz) using $CDCl_3$ as solvent, 300 MHz and 75 MHz, respectively. Mass spectral data were acquired on a Trace GC–MS 2000 ThermoQuest. Melting points were measured on a Büchi 535.

General procedure for the preparation of arylalkynes 2a–q (non-decarboxylative approach, method A). An oven-dried 20 mL Schlenk tube equipped with a magnetic stirring bar and a rubber septum was charged with $Pd(OAc)_2$ (6.7 mg, 30 µmol), tri(*p*-tolyl)phosphine (18.2 mg, 60 µmol). After purging the vessel with alternating vacuum and nitrogen cycles, degassed THF (3 mL), 1,8-diazabicycloundec-7-ene (450 µL, 3.0 mmol), 2-methylbut-3-yn-2-ol (120 µL, 1.24 mmol) and aryl bromides **1a–q** (1.0 mmol) were added via a syringe (solid aryl bromides were added as solution in degassed THF) and the mixture was stirred at 80 °C for 6 h. After cooling to rt the mixture was diluted with water (20 mL) and extracted with AcOEt (3 × 20 mL). Combined organic extracts were washed with H_2O (20 mL), saturated aqueous NaCl (20 mL), dried over $MgSO_4$ and concentrated in vacuum. The crude product was purified by silica gel chromatography (eluant hexane/Et_2O gradient) affording the corresponding products **2a–q**.

General procedure for the preparation of arylalkynes 2a,b, 2d–h and 2j (decarboxylative approach, method B). An oven-dried 20 mL Schlenk tube equipped with a magnetic stirring bar and a rubber septum was charged with $Pd(OAc)_2$ (5.6 mg, 25 µmol), 2-dicyclohexylphosphino-2',6'-dimethoxybiphenyl (15.4 mg, 37.5 µmol) and 4-hydroxy-4-methyl-2-pentynoic acid **6** (80 mg, 0.63 mmol). After purging the vessel with alternating vacuum and nitrogen cycles, a degassed solution of $TBAF{\cdot}3H_2O$ (0.47 g, 1.5 mmol) in THF (3 mL) was added. Aryl bromides **1a**,**b**, **1d–h** and **1j** (0.5 mmol) were added via a syringe (solid aryl bromides were added as solution in degassed THF) and the mixture was stirred at 80 °C for 14 h. After cooling to rt the mixture was diluted with water (20 mL) and extracted with AcOEt (3 × 20 mL). Combined organic extracts were washed with H_2O (20 mL), saturated aqueous NaCl (20 mL), dried over $MgSO_4$ and concentrated in vacuum. The crude product was purified by silica gel chromatography (eluant cyclohexane/Et_2O or EtOAc gradient) affording the corresponding products **2a**,**b**, **2d–h** and **2j**.

General procedure for the preparation of arylalkynes 2i, 2k, 2n, 2o (decarboxylative approach, method C). An oven-dried 20 mL Schlenk tube equipped with a magnetic stirring bar and a rubber septum was charged with $Pd(OAc)_2$ (5.6 mg, 25 µmol), 2-Dicyclohexylphosphino-2′,4′,6′-triisopropylbiphenyl (17.9 mg, 37.5 µmol), and 4-hydroxy-4-methyl-2-pentynoic acid **6** (128 mg, 1.0 mmol). After purging the vessel with alternating *vacuum* and nitrogen cycles, a degassed solution of $TBAF{\cdot}3H_2O$ (0.47 g, 1.5 mmol) in THF (3 mL) was added. Aryl bromides **1i**, **1k**, **1n**, **1o** (0.5 mmol) were added via syringe (solid aryl bromides were added as solution in degassed THF) and the mixture was stirred at 80 °C for 14 h. After cooling to rt the mixture was diluted with water (20 mL) and extracted with AcOEt (3 × 20 mL). Combined organic extracts were washed with H_2O (20 mL), saturated aqueous NaCl (20 mL), dried over $MgSO_4$ and concentrated in *vacuum*.

The crude product was purified by silica gel chromatography (eluant cyclohexane/Et_2O or EtOAc gradient) affording the corresponding products **2i**, **2k**, **2n**, **2o**.

4-Hydroxy-4-methyl-2-pentynoic acid (6) [CAS-No 50624-25-4]

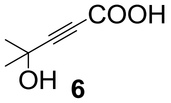

6

Propiolic acid (1.2 mL, 19,5 mmol) was added to a mixture of KOH (6.56 g, 117 mmol) and acetone (20 mL) placed in a ice bath. After stirring overnight at rt, water was added until the solid dissolved. The resulting basic aqueous solutions was washed with Et_2O (2 × 20 mL) and then acified with 1 M HCl. The resulting acidic aqueous phase was extracted with EtOAc (3 × 30 mL) and the collected organic extracts were washed with HCl (1 M, 20 mL), brine (20 mL) and dried over $MgSO_4$. Solvents were removed in vacuum resulting a brown oil. The product slowly crystallized after the addition of a 7:3 CH_2Cl_2/cyclohexane mixture to afford 1.32 g (53% yield) of pale yellow crystals. Alternatively, the crude reaction mixture was purified by silica-gel chromatography (eluant pentane/Et_2O in gradient from 8:2 to 5:5) to obtain 1.85 g (74% yield) as a colorless solid; mp 90-91 °C. ^{1}H-NMR (300 MHz, benzene-d_6): δ = 5.59 (s, br, 2 H), 1.30 (s, 6 H) ppm; ^{13}C-NMR (75 MHz, $CDC1_3$): δ = 154.0, 91.5, 73.5, 64.2, 30.1 ppm; IR (KBr): $\tilde{\nu}$ = 3412 (s), 2989 (s), 2241 (m), 1703 (s), 1372 (m), 1264 (s), 1164 (m), 941 (m) cm^{-1}.

2-Methyl-4-(3-aminophenyl)-3-butyn-2-ol (2a) [CAS-No 69088-96-6][1]

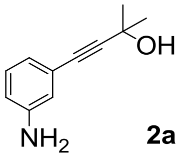

2a

Compound **2a** was synthesized according to the general procedure A from 3-bromoaniline (109 µL, 1 mmol). After column chromatography (SiO_2, ethyl acetate/cyclohexane 3:7) **2a** was isolated as a pale yellow solid (151 mg, 86%); mp 116-118 °C.

Compound **2a** was also prepared according to the general procedure B from 3-bromoaniline (54 µL, 0.5 mmol). After column chromatography (SiO_2, ethyl acetate/cyclohexane 3:7) **2a** was obtained as a pale yellow solid (69.2 mg, 79%); mp 116-118 °C. ^{1}H-NMR (300 MHz, CDC1$_3$): δ = 7.08 (t, *J* = 7.8 Hz, 1 H), 6.82 (dt, *J* = 7.7, 1.2 Hz, 1 H), 6.74 (m, 1 H), 6.63 (ddd, *J* = 8.1, 2.4, 1.0 Hz, 1 H), 3.51 (s, br, 2 H), 2.29 (s, br, 1 H), 1.60 (s, 6 H) ppm; ^{13}C-NMR (75 MHz, CDC1$_3$): δ = 146.2, 129.2, 123.4, 122.0, 117.9, 115.3, 93.2, 82.3, 65.6, 31.5 ppm; MS (70 eV), m/z (%): 175 (18) [M^+], 160 (38), 130 (21), 117 (32), 89 (39), 43 (100).

2-Methyl-4-(3-acetylaminophenyl)-3-butyn-2-ol (2b) [CAS-No 104581-30-8][2]

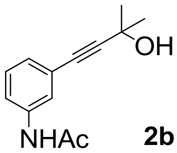

2b

Compound **2b** was synthesized according to the general procedure A from N-(3-bromophenyl)acetamide (214 mg, 1.0 mmol). After column chromatography (SiO_2, ethyl acetate/cyclohexane 3:7) **2b** was isolated as a pale yellow solid (207 mg, 95%); mp 135-136 °C.

Compound **2b** was also prepared according to the general procedure B from N-(3-bromophenyl)acetamide (107 mg, 0.5 mmol). After column chromatography (SiO_2, ethyl acetate/cyclohexane 3:7) **2b** was obtained as a pale yellow solid (86.9 mg, 80%); mp 135-136 °C. ^{1}H NMR (300 MHz, $CDCl_3$): δ = 8.10 (s, br, 1 H), 7.58 (s, 1 H), 7.40 (d, *J* = 7.9 Hz, 1 H), 7.16 (t, *J* = 7.8 Hz, 1 H), 7.08 (d, *J* = 7.5 Hz, 1 H), 3.29 (s, br, 1 H), 2.14 (s, 3 H), 1.57 (s, 6 H) ppm; ^{13}C-NMR (75 MHz, CDC1$_3$): δ = 169.1, 137.9, 128.7, 127.4, 123.3, 123.0,

120.0, 94.1, 81.6, 65.4, 31.4, 24.4 ppm; MS (70 eV), m/z (%): 217 (41) [M^+], 202 (60), 199 (71), 157 (100), 117 (91), 89 (22).

4-(3-Methyl-3-hydroxy-1-butynyl)-*N*,*N*-dimethylaniline (2c) [CAS-No 56512-48-2][3]

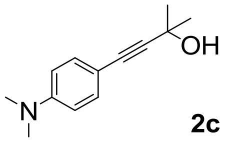

2c

Compound **2c** was synthesized according to the general procedure A from 4-bromo-*N*,*N*-dimethylaniline (200 mg, 1.0 mmol). After column chromatography (SiO_2, diethyl ether/cyclohexane 3:7) **2b** was isolated as a pale yellow solid (148 mg, 73%); mp 83-85 °C. ^{1}H NMR (300 MHz, $CDCl_3$): δ = 7.28 (d, *J* = 8.9 Hz, 2 H), 6.61 (d, *J* = 8.9 Hz, 2 H), 2.95 (s, 6 H), 2.21 (s, 1 H), 1.61 (s, 6 H); ^{13}C-NMR (75 MHz, $CDC1_3$): δ = 150.0, 132.6, 11.7, 109.5, 91.5, 82.9, 65.7, 40.2, 31.6 ppm; MS (70 eV), m/z (%): 203 (12) [M^+], 185 (28), 141 (19), 115 (59), 63 (100).

2-Methyl-4-(3,5-dimethoxyphenyl)-3-butyn-2-ol (2d) [CAS-No 171290-51-0][4]

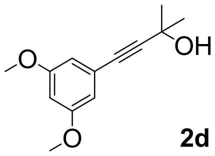

2d

Compound **2d** was synthesized according to the general procedure A from 1-bromo-3,5-dimethoxybenzene (217 mg, 1.0 mmol). After column chromatography (SiO_2, diethyl ether/cyclohexane 2:8) **2d** was isolated as a pale yellow oil (196 mg, 89%).

Compound **2d** was also prepared according to the general procedure B from 1-bromo-3,5-dimethoxybenzene (108 mg, 0.5 mmol). After column chromatography (SiO_2, diethyl ether/cyclohexane 2:8) **2d** was obtained as a pale yellow oil (76.0 mg, 69%). ^{1}H NMR (300 MHz, $CDCl_3$): δ = 6.57 (d, *J* = 2.2 Hz, 2 H), 6.43 (t, *J* = 2.3 Hz, 1 H), 3.77 (s, 6 H), 2.14 (s,

br, 1 H), 1.61 (s, 6 H) ppm; ^{13}C-NMR (75 MHz, CDC1$_3$): δ = 160.4, 124.0, 109.4, 101.8, 93.3, 82.1, 65.6, 55.4, 31.4 ppm; MS (70 eV), m/z (%): 220 (23) [M^+], 202 (52), 128 (60), 115 (100).

2-Methyl-4-(4-methoxy)phenyl-3-butyn-2-ol (2e) [CAS-No 84384-30-5][5]

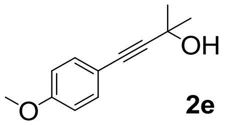

2e

Compound **2e** was synthesized according to the general procedure A from 4-bromoanisole (125 µL, 1.0 mmol). After column chromatography (SiO_2, diethyl ether/cyclohexane 1:9) **2e** was isolated as a pale yellow solid (133 mg, 70%); mp 48-50 °C.

Compound **2e** was also prepared according to the general procedure B from 4-bromoanisole (63 µL, 0.5 mmol). After column chromatography (SiO_2, diethyl ether/cyclohexane 1:9) **2e** was obtained as a pale yellow solid (53.3 mg, 56%); mp 48-50 °C. ^{1}H NMR (300 MHz, $CDCl_3$): δ = 7.34 (d, *J* = 8.9 Hz, 2 H), 6.82 (d, *J* = 8.9 Hz, 2 H), 3.79 (s, 3 H), 2.27 (s, 1 H), 1.60 (s, 6 H) ppm; ^{13}C-NMR (75 MHz, CDC1$_3$): δ = 159.5, 133.0, 114.8, 113.8, 92.4, 81.9, 65.6, 55.2, 31.5 ppm; MS (70 eV), m/z (%): 175 (18) [M^+], 190 (38), 175 (100), 172 (94), 157 (30), 132 (35), 128 (33), 89 (17).

2-Methyl-4-(2-methylphenyl)-3-butyn-2-ol (2f) [CAS-No 40888-14-0][6]

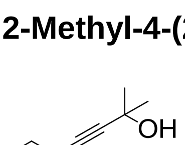

2f

Compound **2f** was synthesized according to the general procedure A from 2-bromotoluene (120 µL, 1.0 mmol). After column chromatography (SiO_2, diethyl ether/cyclohexane 1:9) **2f** was isolated as a pale yellow oil (139 mg, 80%).

Compound **2f** was also prepared according to the general procedure B from 2-bromotoluene (60 μL, 0.5 mmol). After column chromatography (SiO_2, diethyl ether/cyclohexane 1:9) **2f** was obtained as a pale yellow oil (68.0 mg, 78%). ^{1}H NMR (300 MHz, $CDCl_3$): δ = 7.38 (d, *J* = 7.5 Hz, 1 H), 7.09-7.24 (m, 3 H), 2.42(s, 3 H), 2.26 (s, br, 1 H), 1.65(s, 6 H) ppm; ^{13}C-NMR (75 MHz, $CDC1_3$): δ = 140.1, 131.8, 129.3, 128.2, 125.4, 122.4, 97.9, 81.0, 65.7, 31.6, 20.5 ppm; MS (70 eV), m/z (%): 174 (36) [M^+], 159 (100), 128 (11), 115 (42), 91 (15).

2-Methyl-4-(3-methylphenyl)-3-butyn-2-ol (2g) [CAS-No 63079-66-3][7]

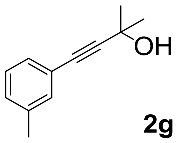

2g

Compound **2g** was synthesized according to the general procedure A from 3-bromotoluene (121 μL, 1.0 mmol). After column chromatography (SiO_2, diethyl ether/cyclohexane 1:9) **2g** was isolated as a pale yellow oil (155 mg, 89%).

Compound **2g** was also prepared according to the general procedure B from 3-bromotoluene (61 μL, 0.5 mmol). After column chromatography (SiO_2, diethyl ether/cyclohexane 1:9) **2g** was obtained as a pale yellow oil (71.4 mg, 82%). ^{1}H NMR (300 MHz, $CDCl_3$): δ = 6.99-7.25 (m, 4 H), 2.32 (s, 3 H), 2.26 (s, br, 1 H), 1.62 (s, 6 H) ppm. ^{13}C-NMR (75 MHz, $CDC1_3$): δ = 137.9, 132.2, 129.1, 128.6, 128.1, 122.5, 93.4, 82.2, 65.5, 31.5, 21.1 ppm; MS (70 eV), m/z (%): 174 (24) [M^+], 159 (100), 131 (9), 115 (27), 91 (12).

2-Methyl-4-(4-methylphenyl)-3-butyn-2-ol (2h) [CAS-No 79756-91-5][7]

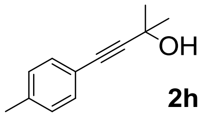

2h

Compound **2h** was synthesized according to the general procedure A from 4-bromotoluene (122 μL, 1.0 mmol). After column chromatography (SiO_2, diethyl

ether/cyclohexane 1:9) **2h** was isolated as a pale yellow solid (151 mg, 87%); mp 49-51 °C.

Compound **2h** was also prepared according to the general procedure B from 4-bromotoluene (62 µL, 0.5 mmol). After column chromatography (SiO_2, diethyl ether/cyclohexane 1:9) **2h** was obtained as a pale yellow solid (151 mg, 87%); mp 49-51 °C. ^{1}H NMR (300 MHz, $CDCl_3$): δ = 7.31 (d, *J* = 8.1 Hz, 2 H), 7.10 (d, *J* = 8.5 Hz, 2 H), 2.34 (s, 3 H), 2.22 (s, 1 H), 1.61 (s, 6 H) ppm; ^{13}C-NMR (75 MHz, $CDC1_3$): δ = 138.2, 131.5, 128.9, 119.6, 93.1, 82.2, 65.6, 31.5, 21.4 ppm; MS (70 eV), *m/z* (%): 174 (29) [M^+], 159 (100), 131 (11), 115 (27), 91 (12).

2-Hydroxy-2-methyl-4-phenyl-3-butyne (2i) [CAS-No 1719-19-3][8]

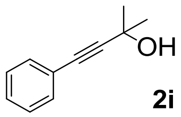

2i

Compound **2i** was synthesized according to the general procedure A from bromobenzene (105 µL, 0.5 mmol). After column chromatography (SiO_2, diethyl ether/cyclohexane 2:8) **2i** was isolated as a pale yellow solid (150 mg, 94%); mp 49-51 °C.

Compound **2i** was also prepared according to the general procedure C from bromobenzene (53 µL, 0.5 mmol). After column chromatography (SiO_2, diethyl ether/cyclohexane 1:9) **2i** was obtained as a pale yellow solid (54.5 mg, 68%); mp 49-51 °C. ^{1}H NMR (300 MHz, $CDCl_3$): δ = 7.40-7.43 (m, 2 H), 7.27-7.32 (m, 3 H), 2.33 (s, br, 1 H), 1.62 (s, 6 H) ppm; ^{13}C-NMR (75 MHz, $CDC1_3$): δ = 131.6, 128.2, 128.2, 122.7, 93.8, 82.1, 65.6, 31.4 ppm; MS (70 eV), m/z (%):160 (21) [M^+], 145 (100), 129 (7), 115 (19).

2-Methyl-4-(naphthalen-1-yl)-3-butyn-2-ol (2j) [CAS-No 40888-18-4][9]

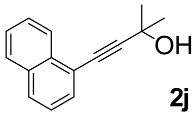

2j

Compound **2j** was synthesized according to the general procedure A from 1-bromonaphthalene (140 µL, 1.0 mmol). After column chromatography (SiO_2, diethyl ether/cyclohexane 2:8) **2j** was isolated as a yellow oil (201 mg, 95%).

Compound **2j** was also prepared according to the general procedure B from 1-bromonaphtalene (70 µL, 0.5 mmol). After column chromatography (SiO_2, diethyl ether/cyclohexane 2:8) **2j** was obtained as a yellow oil (55.7 mg, 53%). ^{1}H NMR (300 MHz, $CDCl_3$): δ = 8.30 (d, *J* = 8.1 Hz, 1 H), 7.83 (t, *J* = 8.4 Hz, 2 H), 7.66 (dd, J = 7.2, 1.1 Hz, 1 H), 7.49-7.60 (m, 2 H), 7.41 (m, 1 H), 2.27 (s, br, 1 H), 1.74 (s, 6 H) ppm; ^{13}C-NMR (75 MHz, $CDC1_3$): δ = 133.2, 133.1, 130.3, 128.7, 128.2, 126.7, 126.3, 126.0, 125.1, 120.3, 98.8, 80.2. 65.9, 31.6 ppm; MS (70 eV), m/z (%): 210 (59) [M^+], 195 (89), 192 (56), 176 (22), 165 (27), 152 (100).

2-Methyl-4-(3-chlorophenyl)-3-butyn-2-ol (2k)[10]

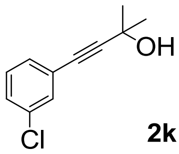

2k

Compound **2k** was synthesized according to the general procedure A from 1-bromo-3-chlorobenzene (117 µL, 1.0 mmol). After column chromatography (SiO_2, diethyl ether/cyclohexane 1:9) **2k** was isolated as a pale yellow oil (187 mg, 96%).

Compound **2k** was also prepared according to the general procedure C from 1-bromo-3-chlorobenzene (59 µL, 0.5 mmol). After column chromatography (SiO_2, diethyl ether/cyclohexane 1:9) **2k** was obtained as a pale yellow oil (24.3 mg, 25%). ^{1}H NMR (300 MHz, $CDCl_3$): δ = 7.40-7.41 (m, 1 H), 7.19-7.31 (m, 3 H), 2.08 (s, 1 H), 1.61 (s, 6 H) ppm; ^{13}C-NMR (75 MHz, $CDC1_3$): δ = 134.1, 131.5, 129.7, 129.4, 128.5, 124.4, 94.9, 80.8, 65.6, 31.4 ppm; MS (70 eV), m/z (%): 195 (4) [M^+], 176 (10), 136 (33), 111 (70), 63 (100).

2-Methyl-4-(4-Fluorophenyl)-3-butyn-2-ol (2l)[11]

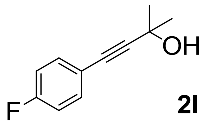

2l

Compound **2l** was synthesized according to the general procedure A from 1-bromo-4-fluorobenzene (110 µL, 1.0 mmol). After column chromatography (SiO_2, diethyl ether/cyclohexane 1:9) **2l** was isolated as a pale yellow oil (172 mg, 96%). ^{1}H NMR (300 MHz, $CDCl_3$): δ = 7.35-7.40 (m, 2 H), 6.95-7.01 (m, 2 H), 2.30 (s, 1 H), 1.60 (s, 6 H); ^{13}C-NMR (75 MHz, $CDC1_3$): δ = 162.4 (d, *J* = 249.6 Hz), 133.5 (d, *J* = 8.3 Hz), 118.8 (d, *J* = 3.4 Hz), 115.5 (d, *J* = 22.1 Hz), 93.4 (d, *J* = 1.4 Hz), 81.0, 65.5, 31.4 ppm; MS (70 eV), m/z (%): 178 (10) [M^+], 159 (30), 133 (17), 87 (29), 87 (29), 63 (100).

2-Methyl-4-(4-(trifluoromethyl)phenyl)-3-butyn-2-ol (2m)[5]

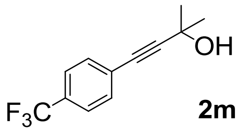

2m

Compound **2m** was synthesized according to the general procedure A from 1-bromo-4-(trifluoromethyl)benzene (140 µL, 1.0 mmol). After column chromatography (SiO_2, diethyl ether/cyclohexane 1:9) **2m** was isolated as a colourless solid (203 mg, 89%); mp 51-52°C. ^{1}H NMR (300 MHz, $CDCl_3$): δ = 7.55 (d, *J* = 8.5 Hz, 2 H), 7.50 (d, *J* = 8.7 Hz, 2 H), 2.22 (s, 1 H), 1.63 (s, 6 H); ^{13}C-NMR (75 MHz, $CDC1_3$): δ = 131.8, 130.0 (q, *J* = 32.6 Hz), 126.6 (q, *J* = 1.4 Hz), 125.1 (q, *J* = 3.7 Hz), 123.9 (q, *J* = 272.1 Hz), 96.2, 80.9, 65.6, 31.3 ppm; MS (70 eV), m/z (%): 228 (10) [M^+], 213 (18), 151 (28), 115 (22), 87 (42), 69 (100).

2-Methyl-4-(3-pyridinyl)-3-butyn-2-ol (2n) [CAS-No 24202-80-0]:[7]

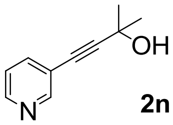

2n

Compound **2n** was synthesized according to the general procedure A from 3-bromopyridine (98 μL, 1.0 mmol). After column chromatography (SiO_2, diethyl ether/cyclohexane 3:7) **2n** was isolated as a pale yellow solid (136 mg, 84%); mp 53-55 °C.

Compound **2n** was also prepared according to the general procedure C from 3-bromopyridine (49 μL, 0.5 mmol). After column chromatography (SiO_2, diethyl ether/cyclohexane 3:7) **2n** was obtained as a pale yellow solid (49.2 mg, 61%); mp 53-55 °C. ^{1}H NMR (300 MHz, $CDCl_3$): δ = 8.73 (dd, *J* = 2.2, 0.8 Hz, 1 H), 8.49 (dd, *J* = 4.8, 1.6 Hz, 1 H), 7.70 (dt, *J* = 7.9, 1.9 Hz, 1 H), 7.21-7.26 (m, 1 H), 3.92 (s, br, 1 H) 1.61 (s, 6 H) ppm; ^{13}C-NMR (75 MHz, $CDC1_3$): δ = 152.1, 148.2, 138.7, 123.1, 120.2, 98.0, 78.4, 65.1, 31.3 ppm; MS (70 eV), m/z (%): 161 (13) [M^+], 146 (100), 130 (4), 118 (10), 104 (20).

2-Methyl-4-(3-nitrophenyl)-3-butyn-2-ol (2o) [CAS-No 33432-52-9][12]

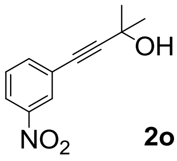

2o

Compound **2o** was synthesized according to the general procedure A from 1-bromo-3-nitrobenzene (202 mg, 1.0 mmol). After column chromatography (SiO_2, diethyl ether/cyclohexane 2:7) **2o** was isolated as a pale yellow solid (157 mg, 77%); mp 46-48 °C.

Compound **2o** was also prepared according to the general procedure C from 1-bromo-3-nitrobenzene (101 mg, 0.5 mmol). After column chromatography (SiO_2, diethyl

ether/cyclohexane 2:7) **2o** was obtained as a pale yellow solid (82.1 mg, 80%); mp 46-48 °C. ^{1}H NMR (300 MHz, $CDCl_3$): δ = 8.25 (t, *J* = 1.8 Hz, 1 H), 8.15 (ddd, *J* = 8.3, 2.2, 1.0 Hz, 1 H), 7.71 (dt, *J* = 7.7, 1.2 Hz, 1 H), 7.48 (t, *J* = 8.1 Hz, 1 H), 2.19 (s, br, 1 H), 1.63 (s, 6 H) ppm; ^{13}C-NMR (75 MHz, $CDC1_3$): δ = 148.0, 137.3, 129.3, 126.5, 124.6, 122.9, 96.4, 79.8, 65.6, 31.3; MS (70 eV), m/z (%): 205 (4) [M^+], 190 (100), 144 (15), 129 (9), 115 (16), 101 (10).

Methyl 4-(3-hydroxy-3-methylbut-1-ynyl)benzoate (2p) [CAS-No 33577-98-9][13]

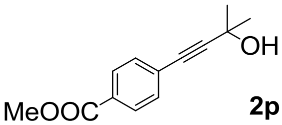

2p

Compound **2p** was synthesized according to the general procedure A from methyl 4-bromobenzoate (215 mg, 1.0 mmol). After column chromatography (SiO_2, diethyl ether/cyclohexane 3:7) **2p** was isolated as a pale yellow solid (157 mg, 72%); mp 75-77 °C. ^{1}H NMR (300 MHz, $CDCl_3$): δ = 7.95 (d, *J* = 8.3 Hz, 2 H), 7.44 (d, *J* = 8.3 Hz, 2 H), 3.90 (s, 3 H), 2.36 (s, 1 H), 1.62 (s, 6 H); ^{13}C-NMR (75 MHz, $CDC1_3$): δ = 166.5, 131.5, 129.5, 129.4, 127.5, 96.8, 81.4, 65.5, 52.2, 31.3 ppm; MS (70 eV), m/z (%): 218 (9) [M^+], 203 (100), 187 (10), 169 (15), 159 (13), 129 (28), 115 (15), 101 (14).

2-Methyl-4-(4'-cyanophenyl)-3-butyn-2-ol (2q) [CAS-No 80151-20-8]**Fehler! Textmarke nicht definiert.**

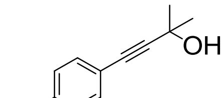

2q

Compound **2q** was synthesized according to the general procedure A from 4-bromobenzonitrile (182 mg, 1.0 mmol). After column chromatography (SiO_2, diethyl ether/cyclohexane 3:7) **2q** was isolated as a pale yellow solid (170 mg, 92%); mp 65-67

°C. ^{1}H NMR (300 MHz, $CDCl_3$): δ = 7.57 (d, *J* = 8.7 Hz, 2 H), 7.46 (d, *J* = 8.7 Hz, 2 H), 2.39 (s, 1 H), 1.61 (s, 6 H); ^{13}C-NMR (75 MHz, $CDC1_3$): δ = 132.1, 131.9, 127.7, 118.3, 111.5, 98.2, 80.5, 65.5, 31.2 ppm; MS (70 eV), m/z (%):185 (11) [M^+], 167 (29), 140 (48), 127 (37), 87 (20), 63 (100).

References

1. Marx, J.; Schumer, F.; Lischewski, R.; Zeckert, K.; Boehme, H. J.; Henning, H. WO Patent WO2005108405 A2, 2005.
2. Arcadi, A.; Cacchi, S.; Marinelli, F. *Tetrahedron* **1985**, *41*, 5121–5132.
3. Umezawa, H.; Okada, S.; Oikawa, H.; Matsuda, H.; Nakanishi, H. *J. Phys. Org. Chem.* **2005**, *18*, 468–472.
4. Dulog, L.; Koerner, B.; Heinze, J.; Yang, J. *Liebigs Annalen* **1995**, *9*, 1663–1672.
5. Kayaki, Y.; Yamamoto, M.; Ikariya, T. *J. Org. Chem*. **2007**, *72*, 647–649.
6. Mori, H.; Matsuo, T.; Yoshioka, Y.; Katsumura, S. *J. Org. Chem.* **2006**, *71*, 9004–9012.
7. Novak, Z.; Szabo, A.; Repasi, J.; Kotschy, A. *J. Org. Chem.* **2003**, *68*, 3327–3329.
8. Zhang, X.; Teo, W.-T.; Chan, P.-W.-H. *Org. Lett.* **2009**, *11*, 4990–4993.
9. Cheng, J.; Sun, Y.; Wang, F.; Guo, M.; Xu, J.-H.; Pan, Y.; Zhang, Z. *J. Org. Chem.* **2004**, *69*, 5428–5432.
10. Zou, H.; Zhou, L.; Li, Y.; Cui, Y.; Zhong, H.; Pan, Z.; Yang, Z.; Quan, J. *J. Med. Chem.* **2010**, *53*, 994–1003.
11. Masaoka, S.; Iwazaki, H. European Patent EP1688424 A1, 2006.
12. Li, J.; Huang P. *Beilstein J. Org. Chem.* **2011**, *7*, 426–431.
13. Li, Y.; Zou, H.; Gong, J.; Xiang, J.; Luo, T.; Quan, J.; Wang, G.; Yang, Z. *Org. Lett.* **2007**, *9*, 4057–4060.

4-Hydroxy-4-methyl-2-pentynoic acid (6)

^{1}H NMR (benzene-d$_6$, 300 MHz)

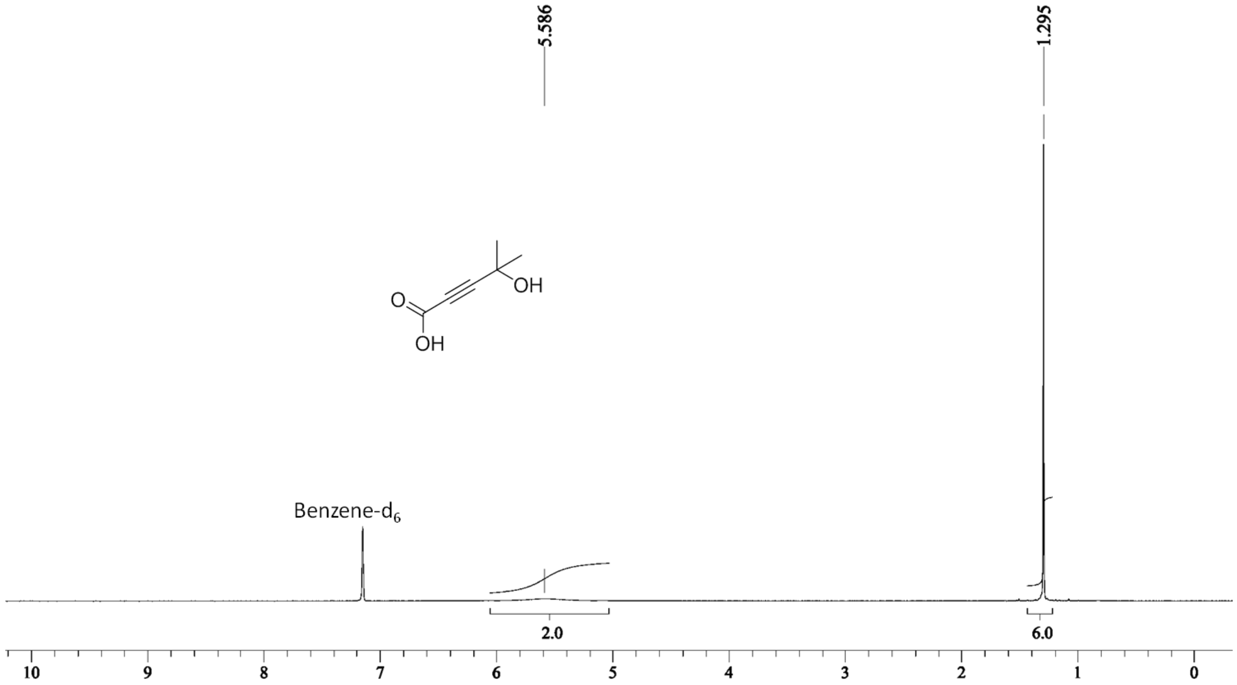

^{13}C NMR (CDCl$_3$, 75 MHz)

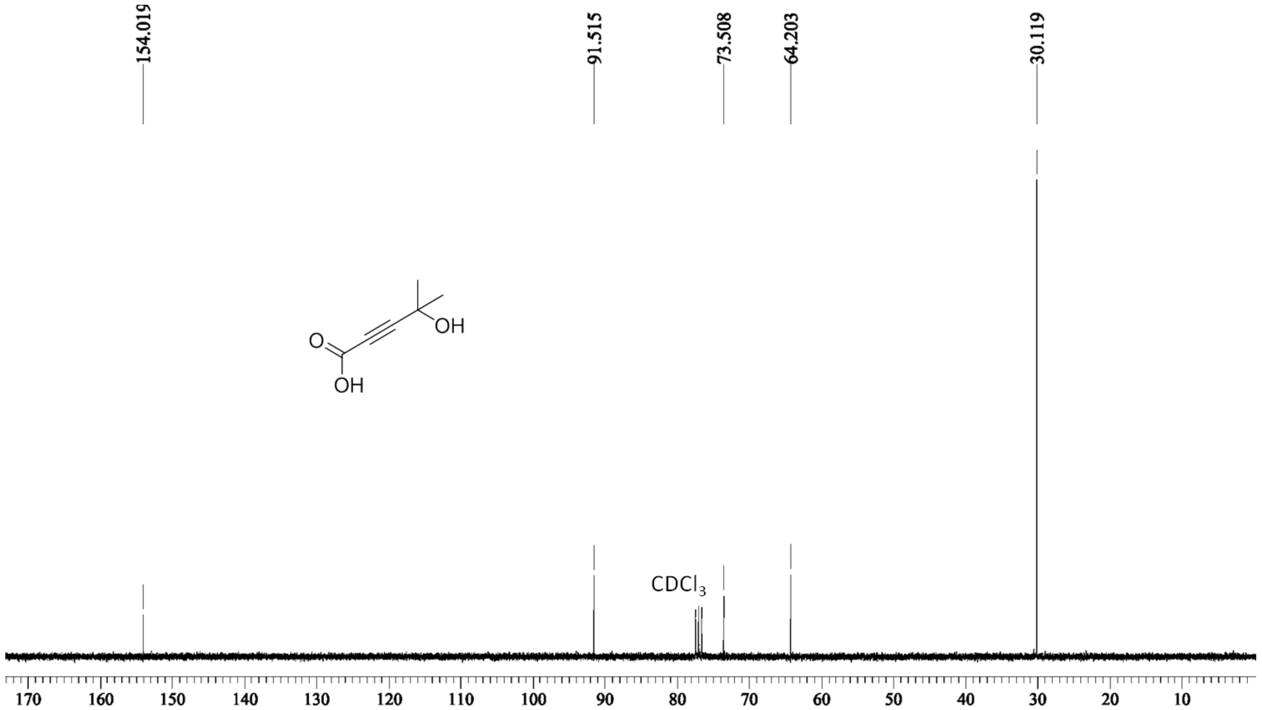

2-Methyl-4-(3-aminophenyl)-3-butyn-2-ol (2a)

^{1}H NMR ($CDCl_3$, 300 MHz)

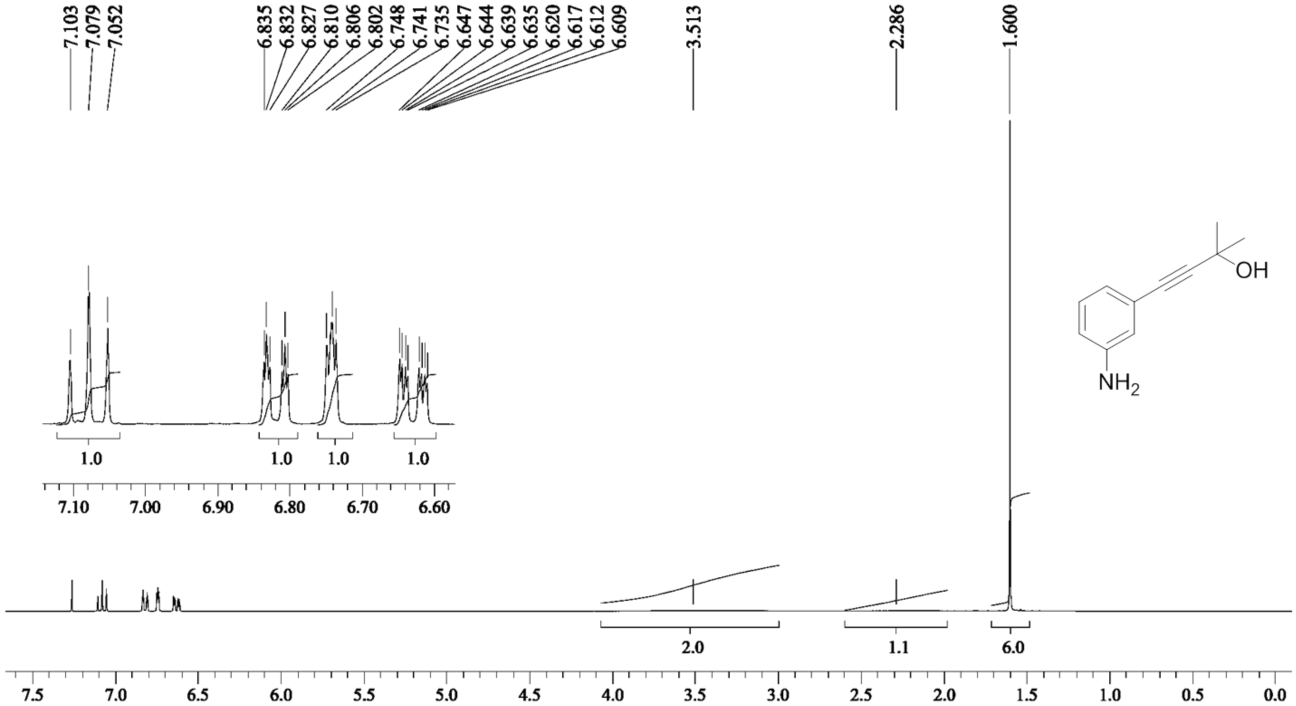

^{13}C NMR ($CDCl_3$, 75 MHz)

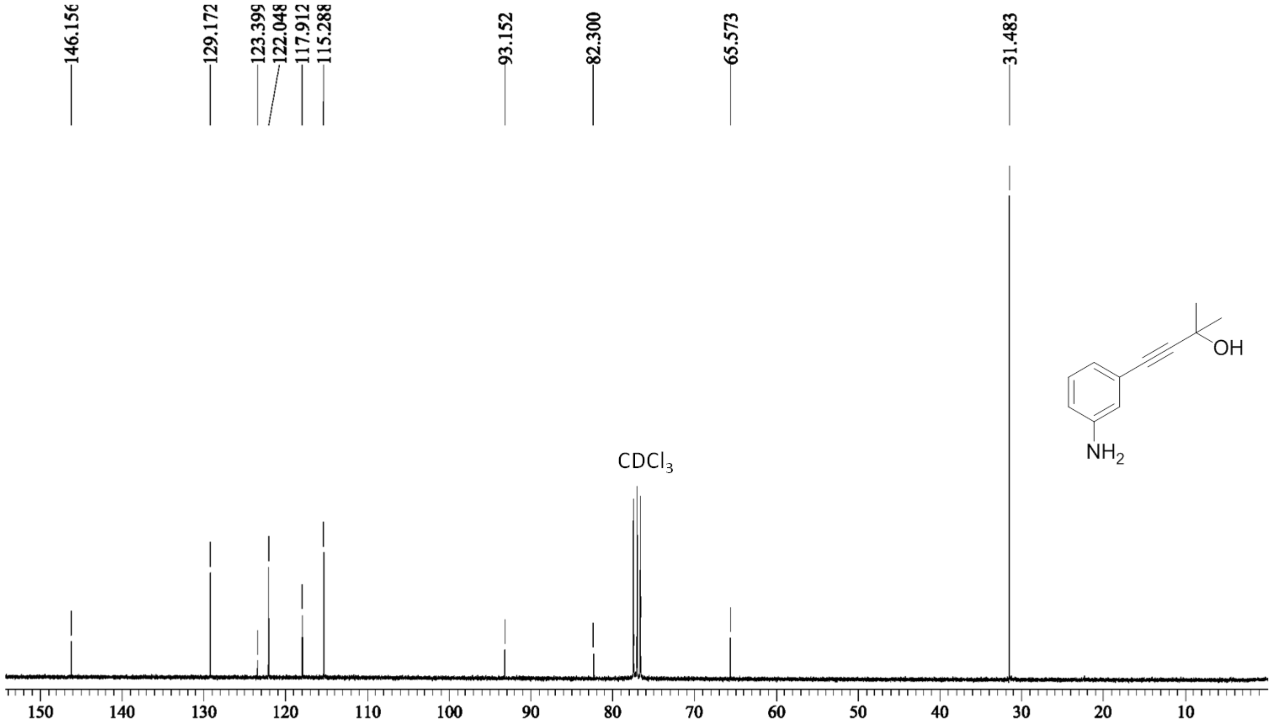

2-Methyl-4-(3-acetylaminophenyl)-3-butyn-2-ol (2b)

^{1}H NMR ($CDCl_3$, 300 MHz)

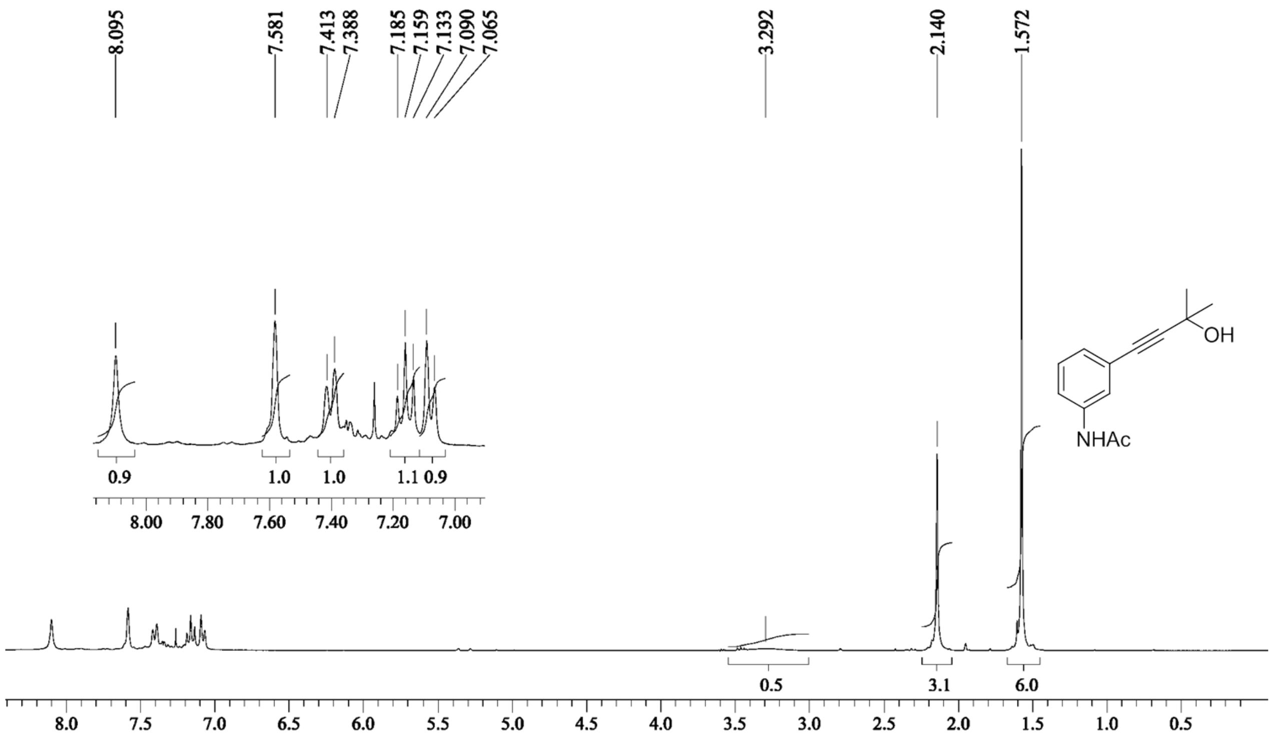

^{13}C NMR ($CDCl_3$, 75 MHz)

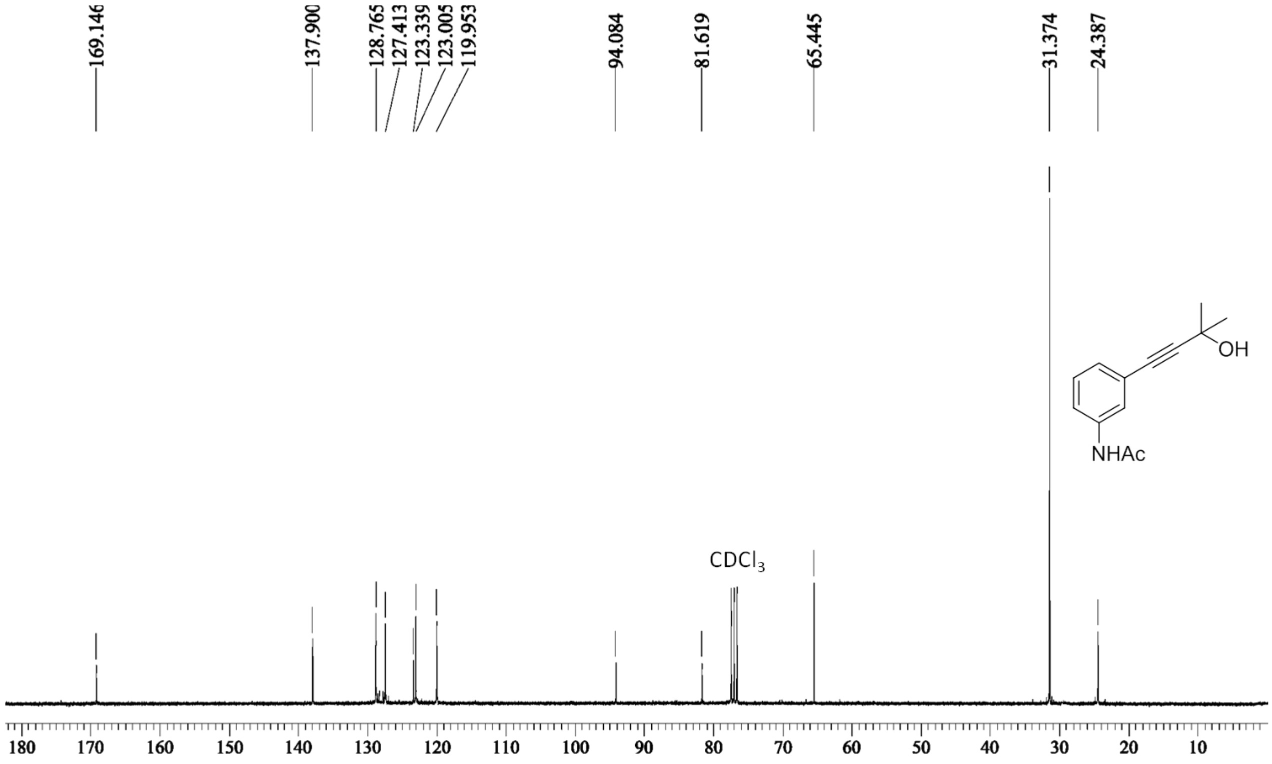

4-(3-Methyl-3-hydroxy-1-butynyl)-N,N-dimethylaniline (2c)

^{1}H NMR ($CDCl_3$, 300 MHz)

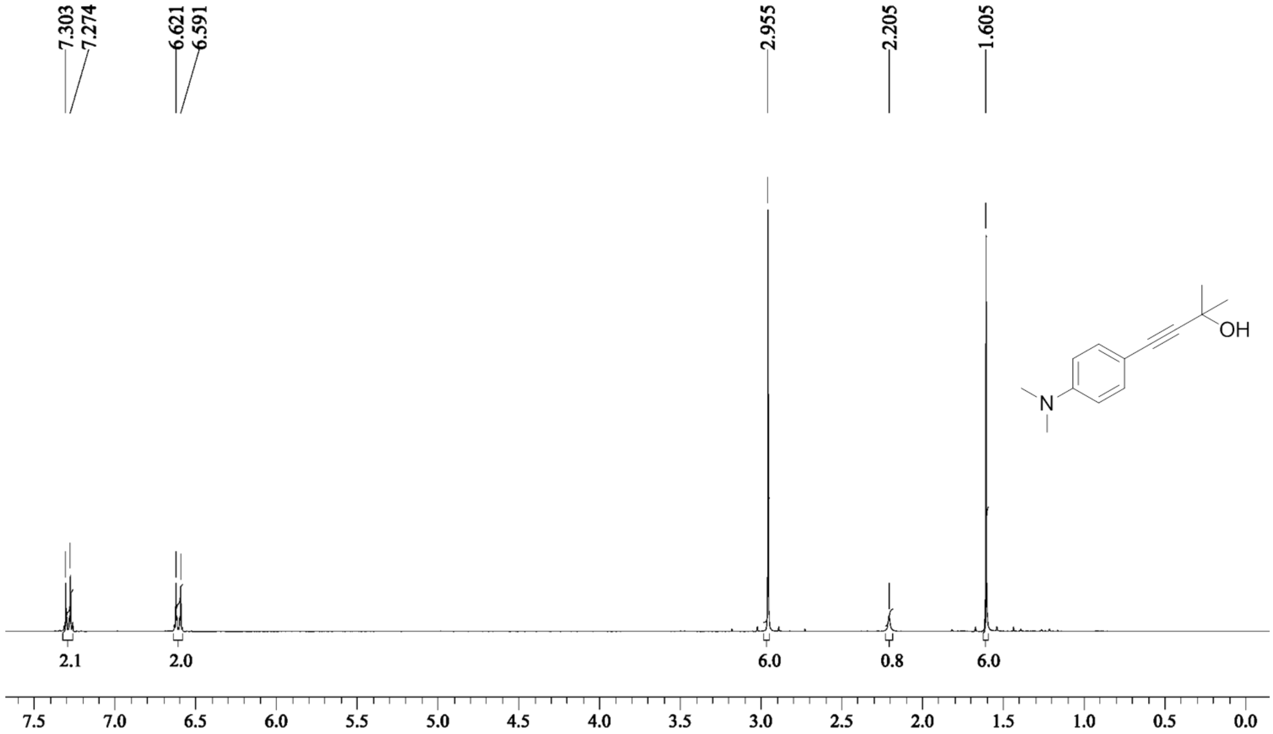

^{13}C NMR ($CDCl_3$, 75 MHz)

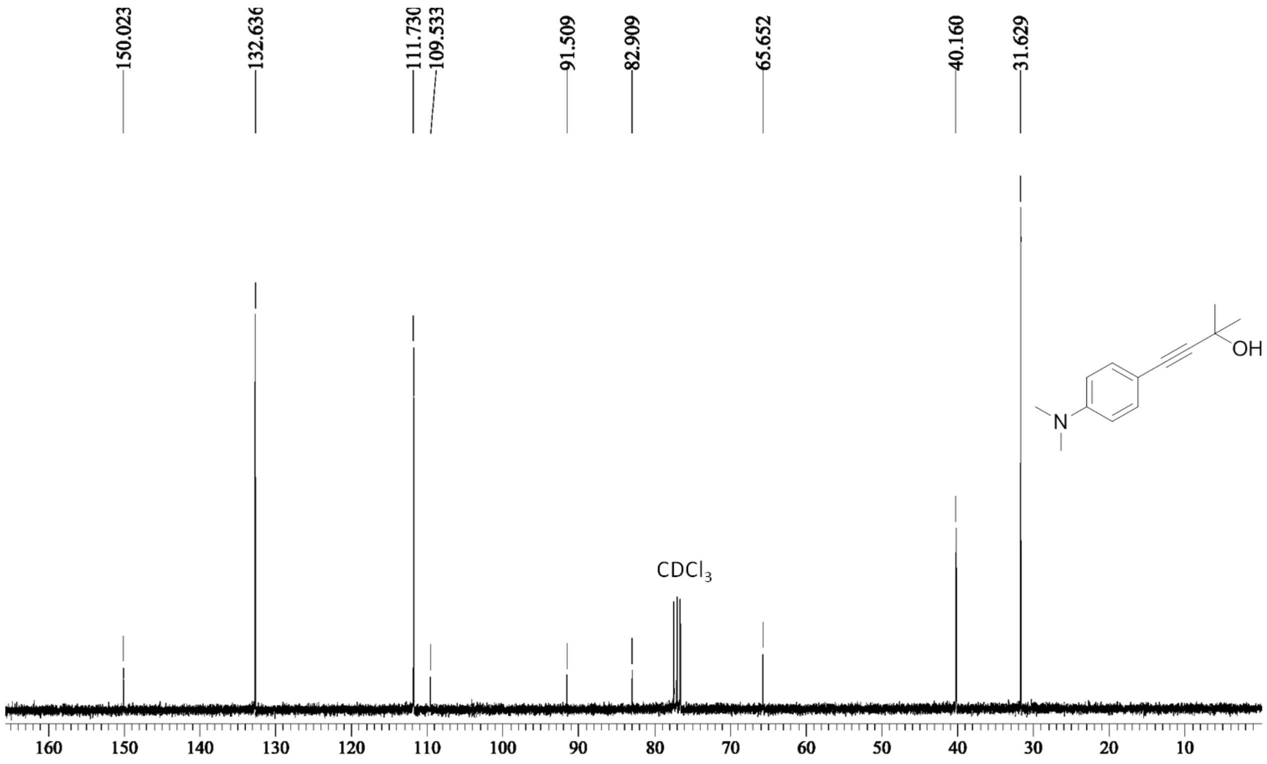

2-Methyl-4-(3,5-methoxyphenyl)-3-butyn-2-ol (2d)

^{1}H NMR ($CDCl_3$, 300 MHz)

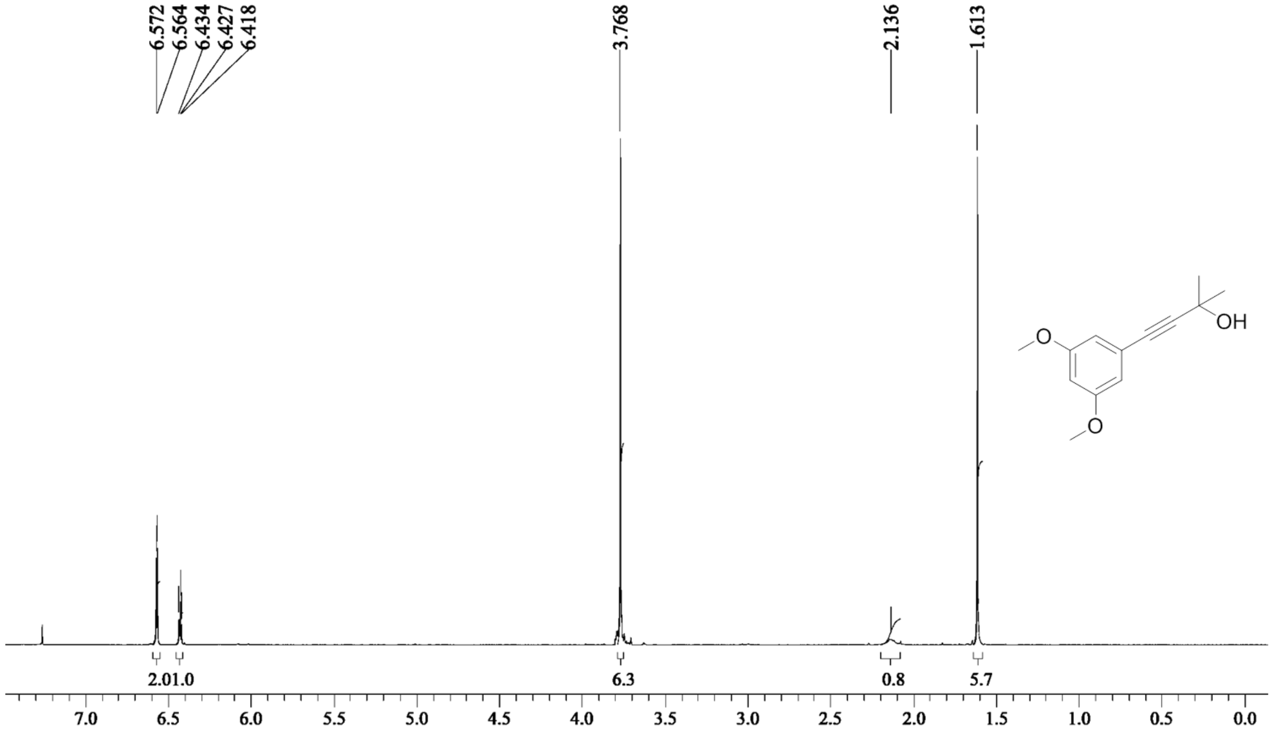

^{13}C NMR ($CDCl_3$, 75 MHz)

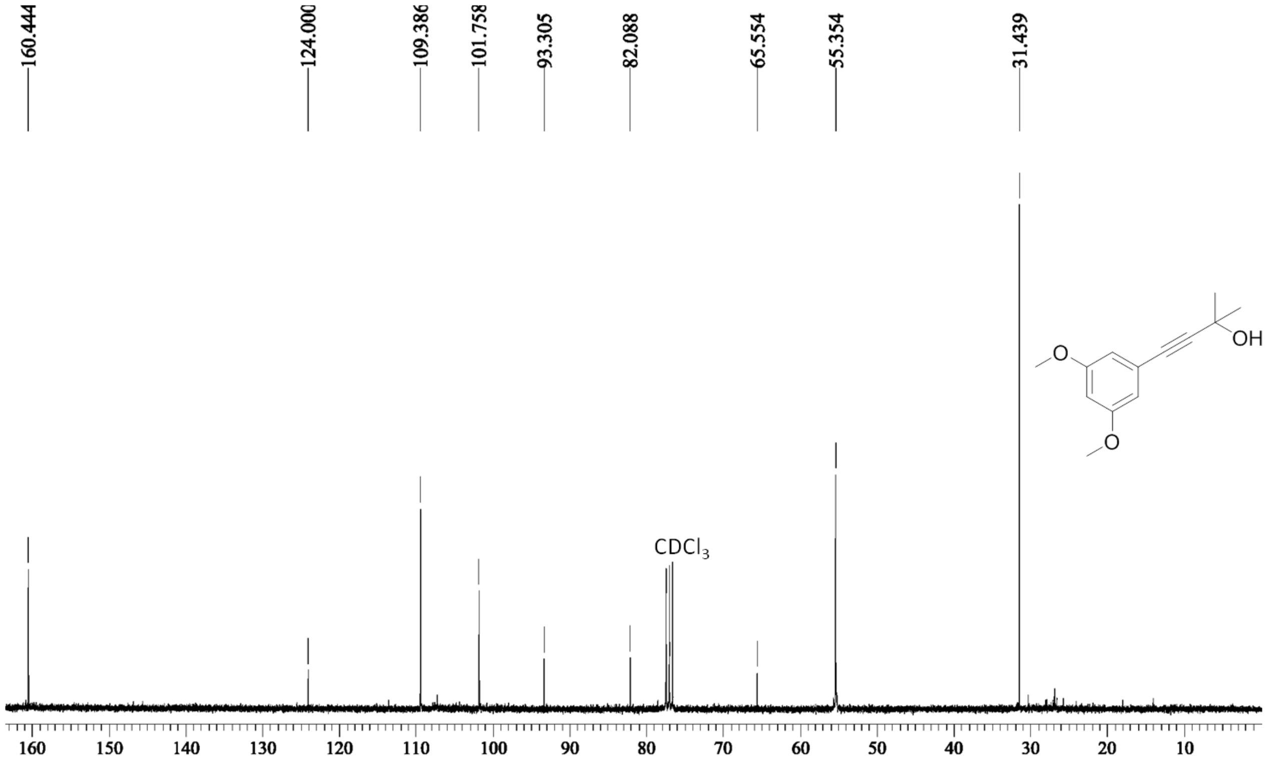

2-Methyl-4-(4-methoxy)phenyl-3-butyn-2-ol (2e)

^{1}H NMR ($CDCl_3$, 300 MHz)

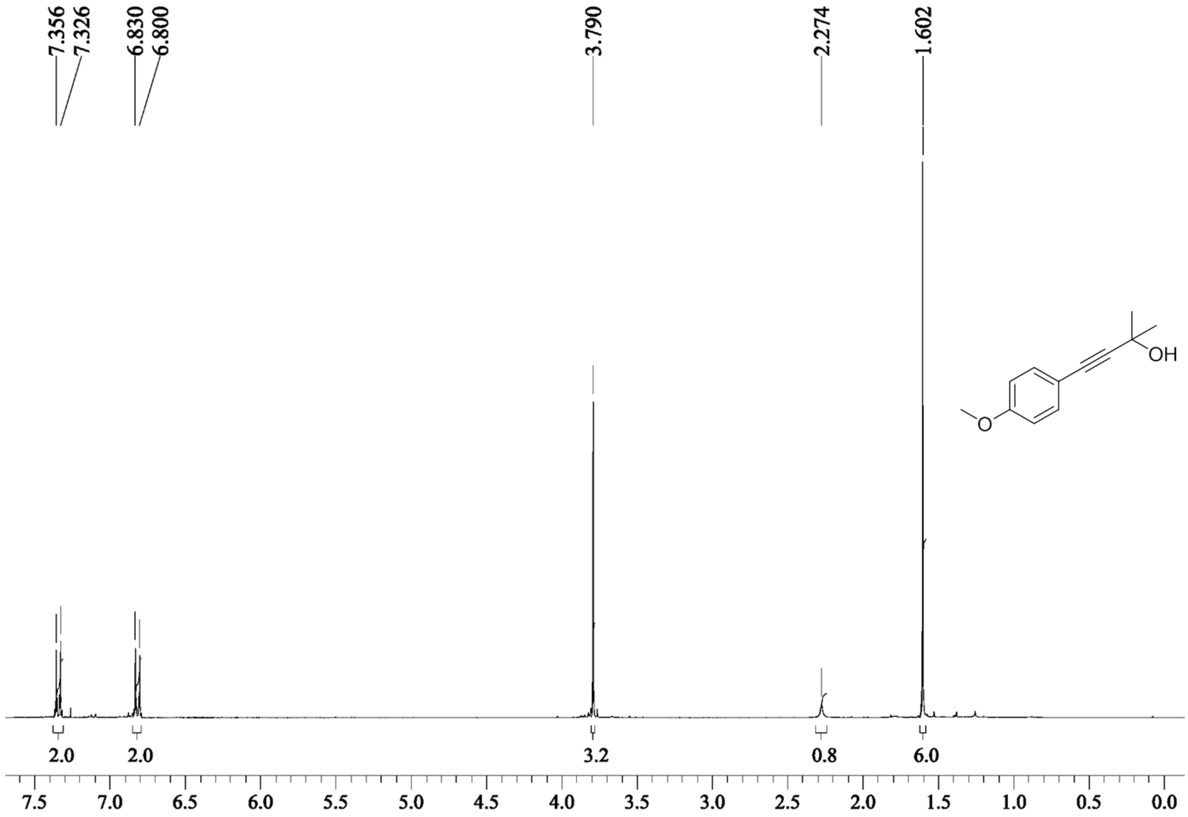

^{13}C NMR ($CDCl_3$, 75 MHz)

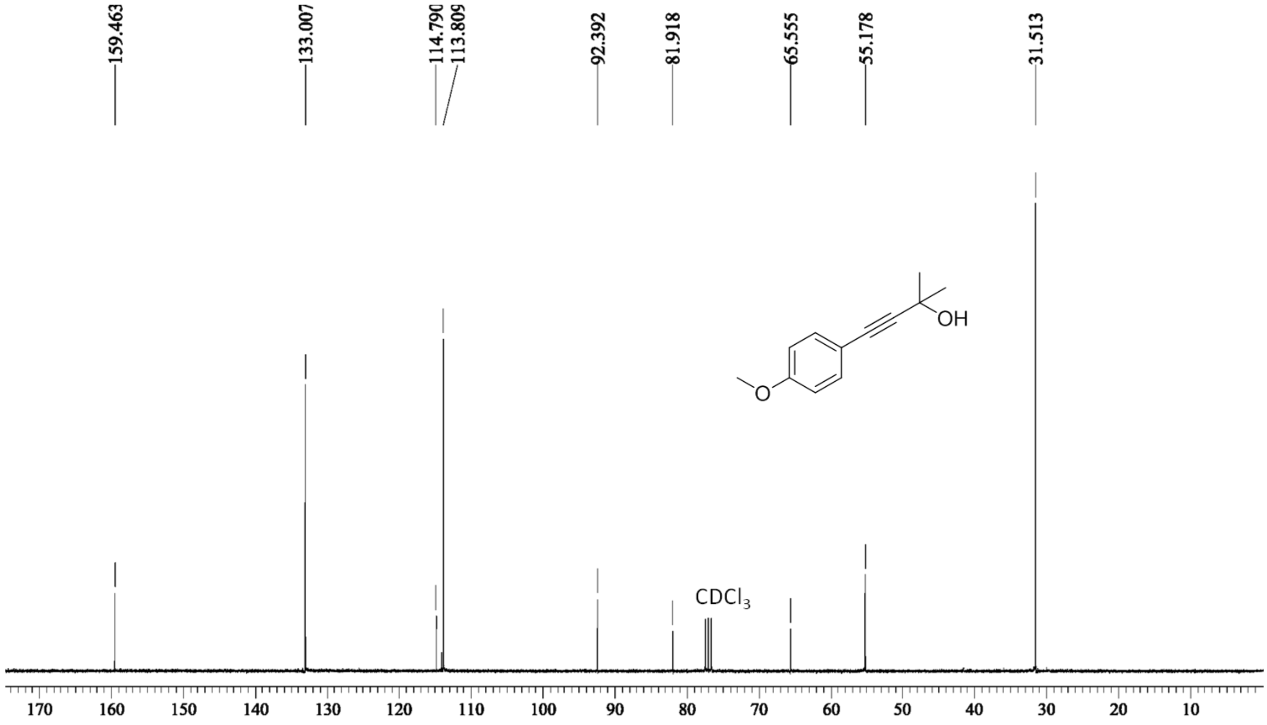

2-Methyl-4-(2-methylphenyl)-3-butyn-2-ol (2f)

^{1}H NMR ($CDCl_3$, 300 MHz)

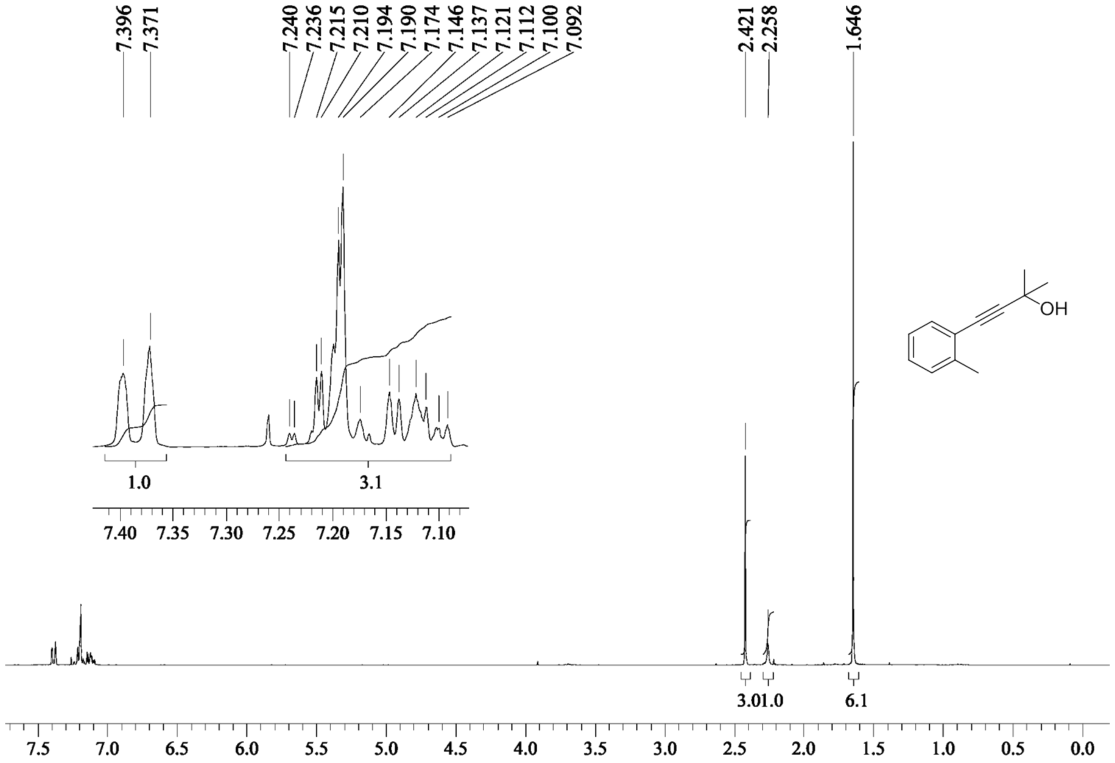

^{13}C NMR ($CDCl_3$, 75 MHz)

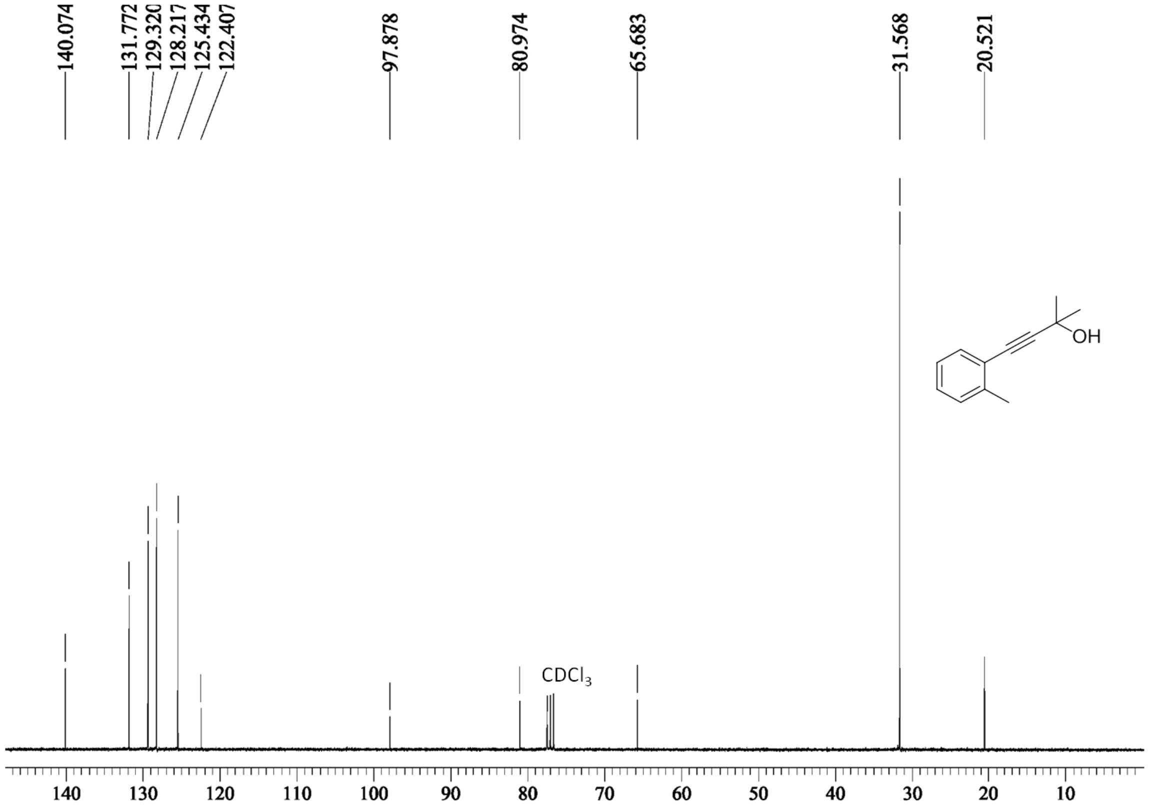

2-Methyl-4-(3-methylphenyl)-3-butyn-2-ol (2g)

^{1}H NMR ($CDCl_3$, 300 MHz)

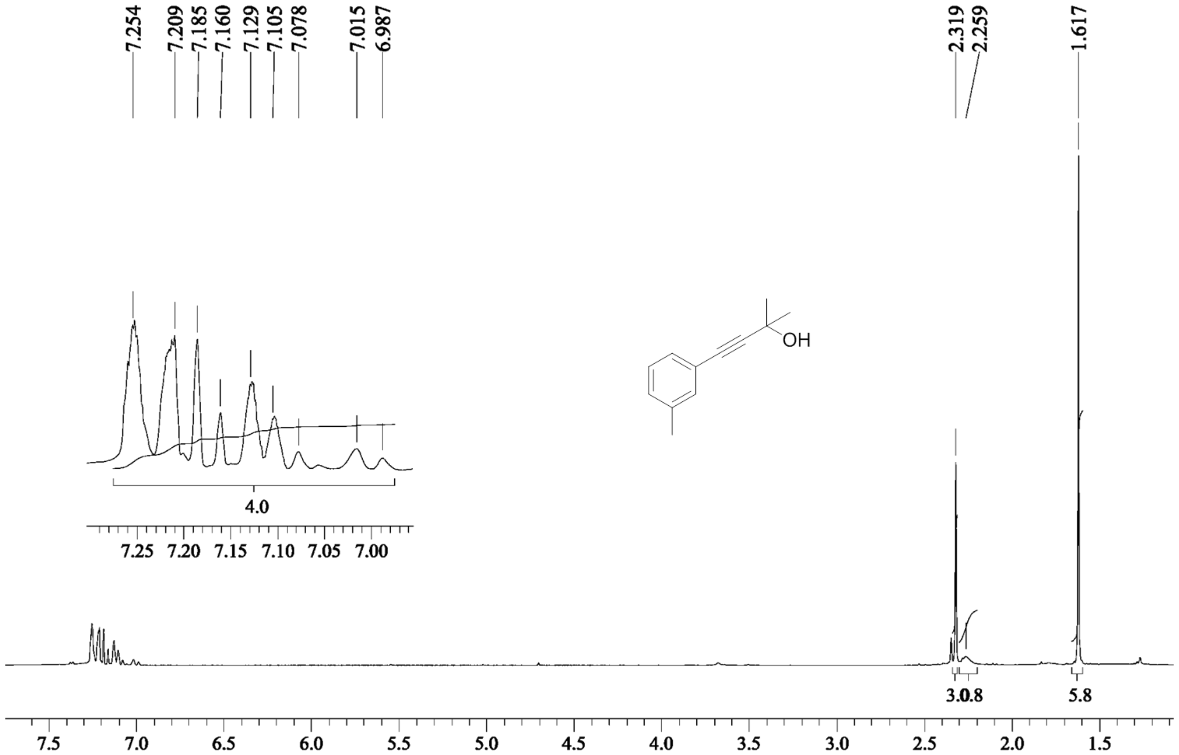

^{13}C NMR ($CDCl_3$, 75 MHz)

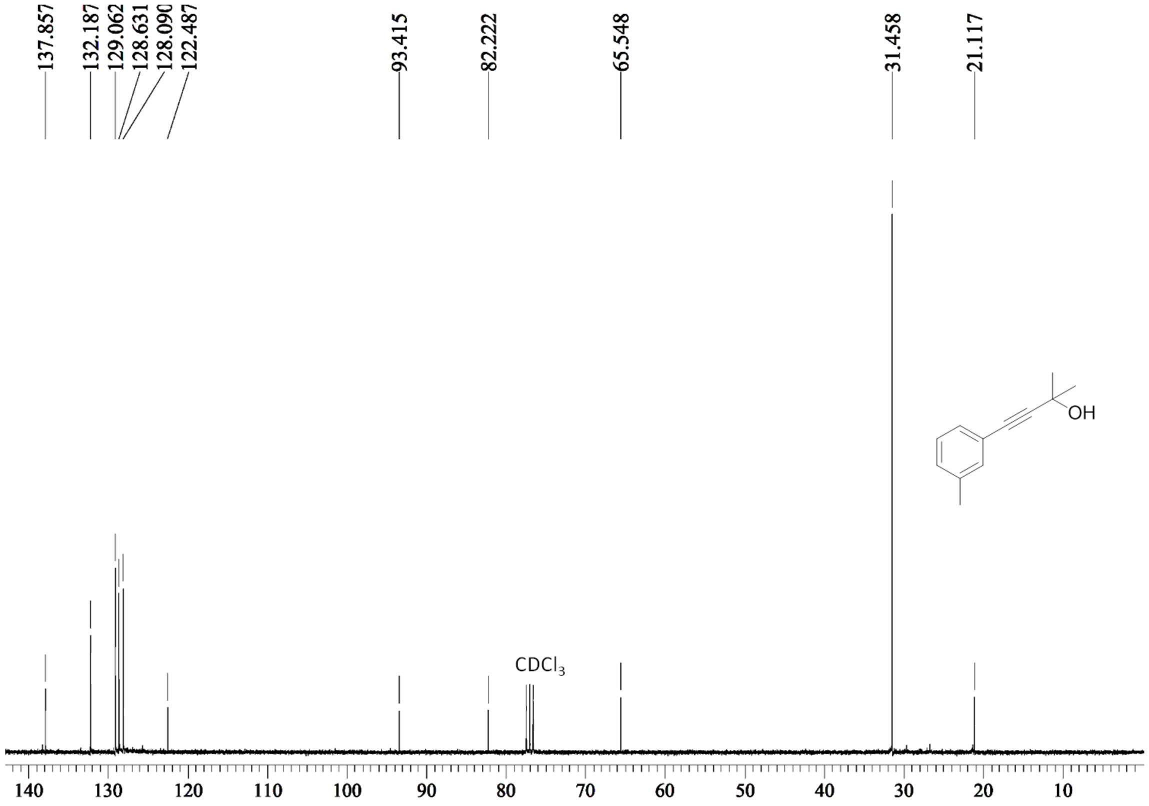

2-Methyl-4-(4-methylphenyl)-3-butyn-2-ol (2h)

^{1}H NMR ($CDCl_3$, 300 MHz)

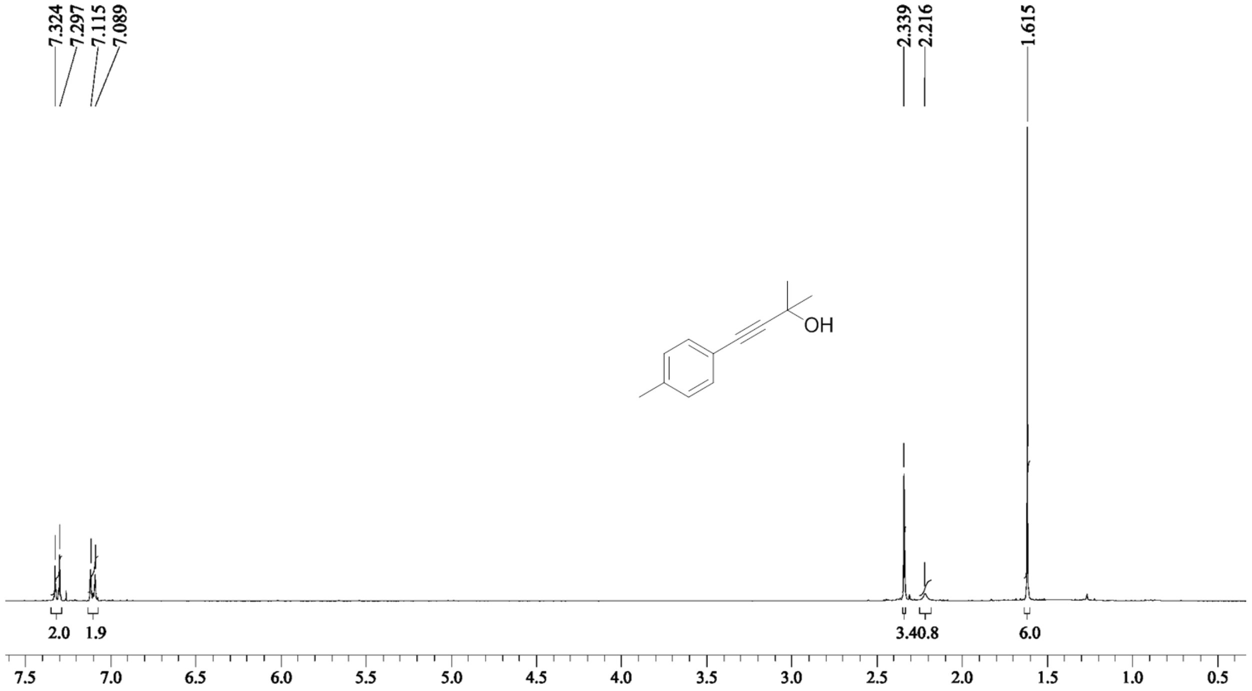

^{13}C NMR ($CDCl_3$, 75 MHz)

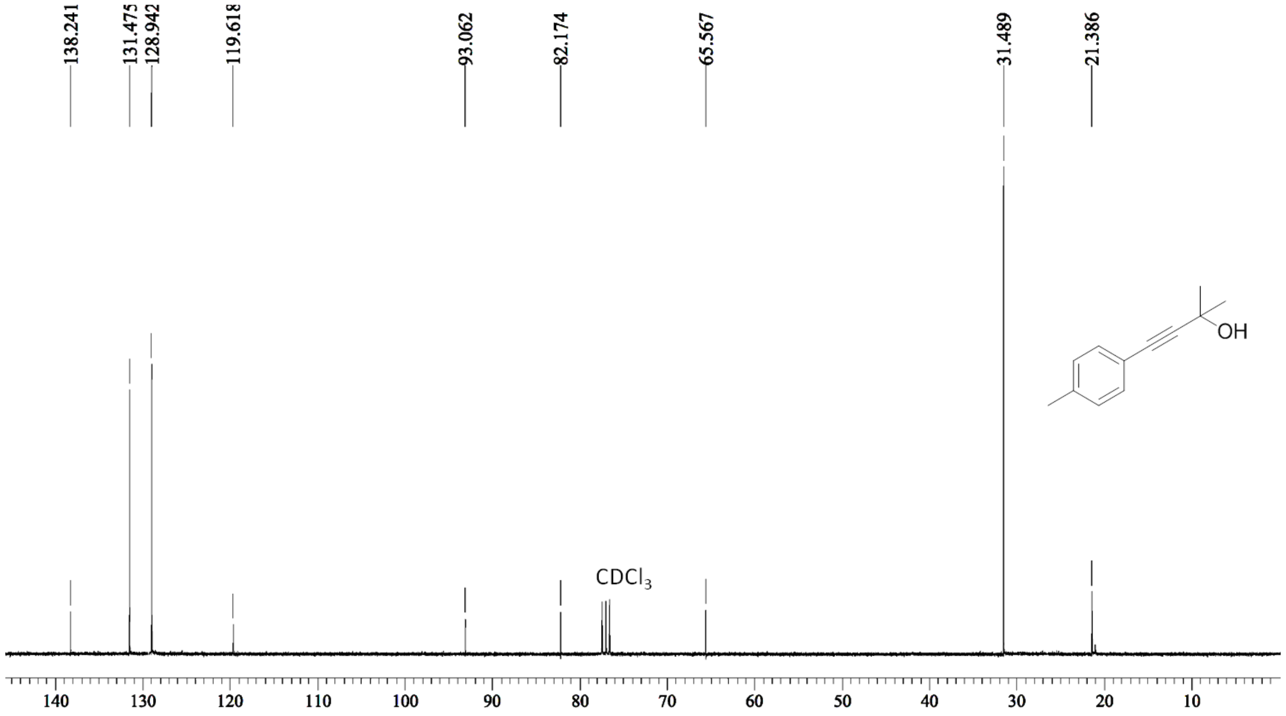

2-Hydroxy-2-methyl-4-phenyl-3-butyne (2i)

^{1}H NMR ($CDCl_3$, 300 MHz)

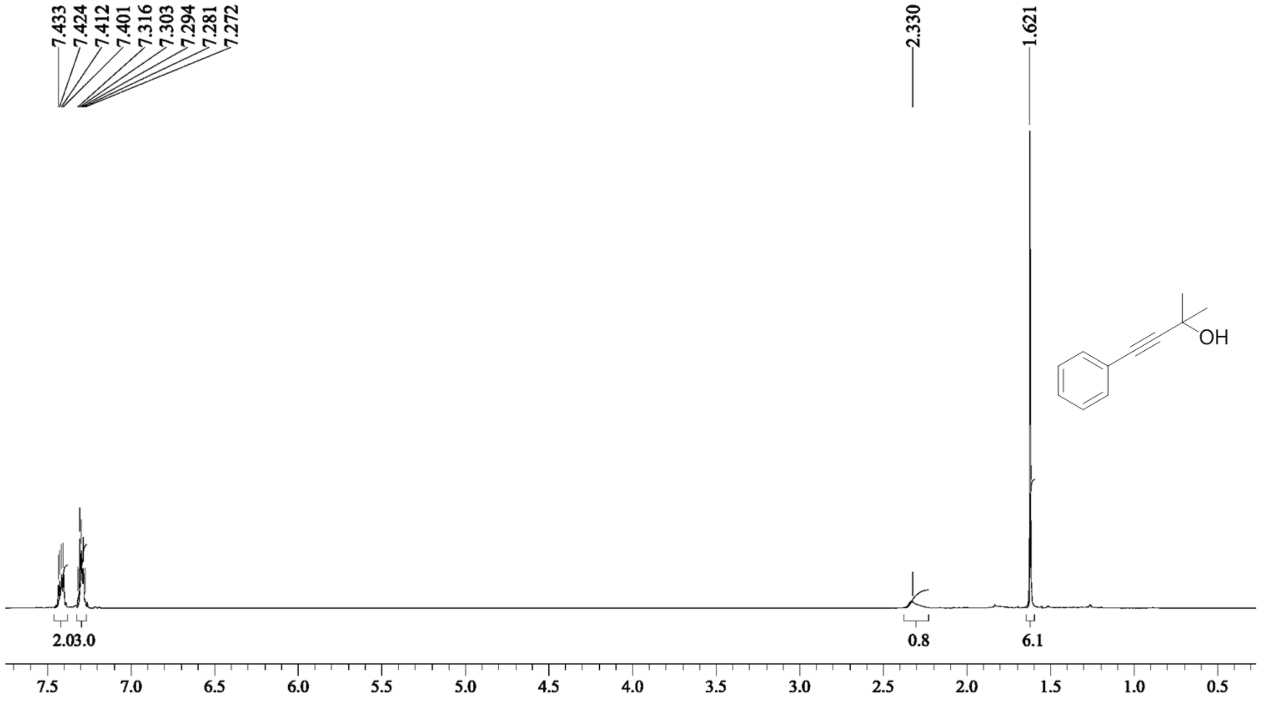

^{13}C NMR ($CDCl_3$, 75 MHz)

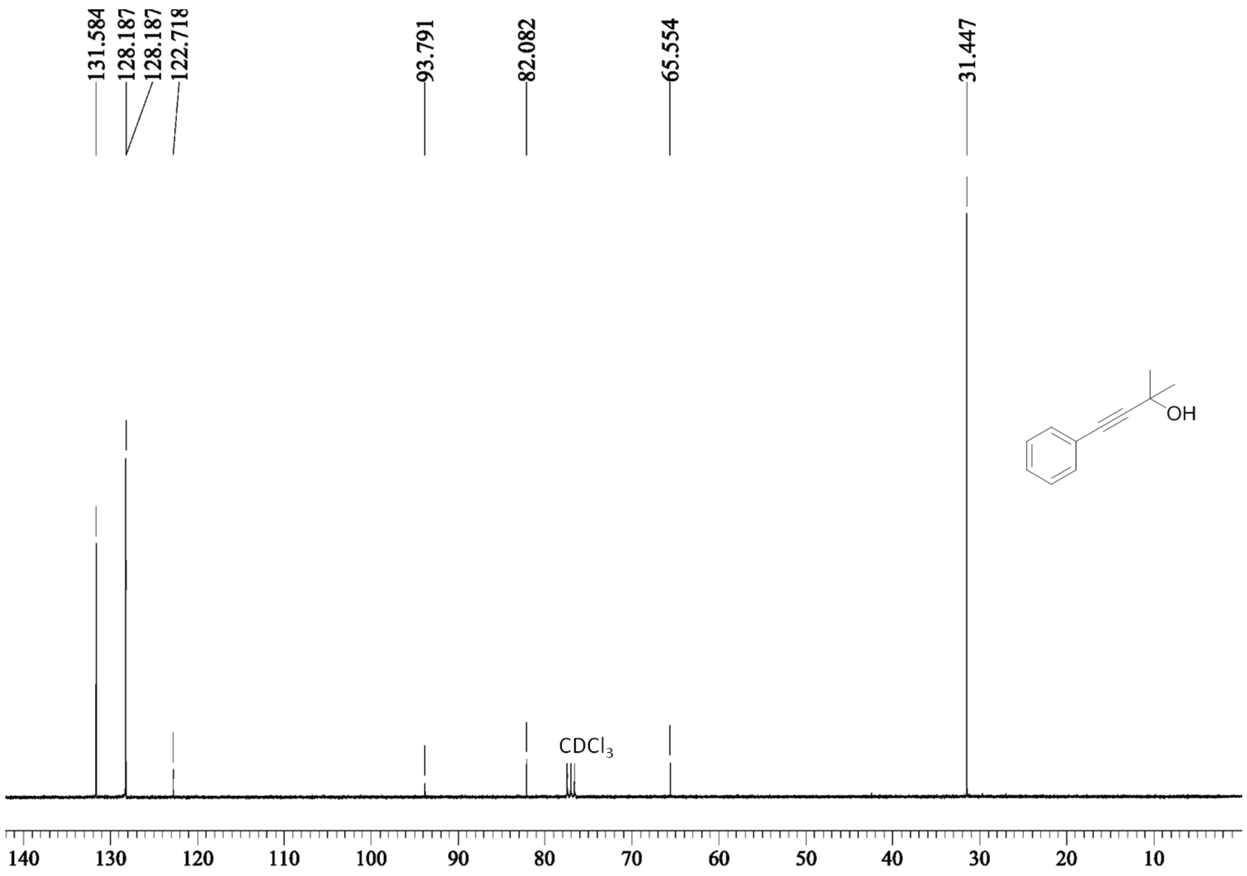

2-Methyl-4-(1-naphthalen-2-yl)-3-butyn-2-ol (2j)

^{1}H NMR ($CDCl_3$, 300 MHz)

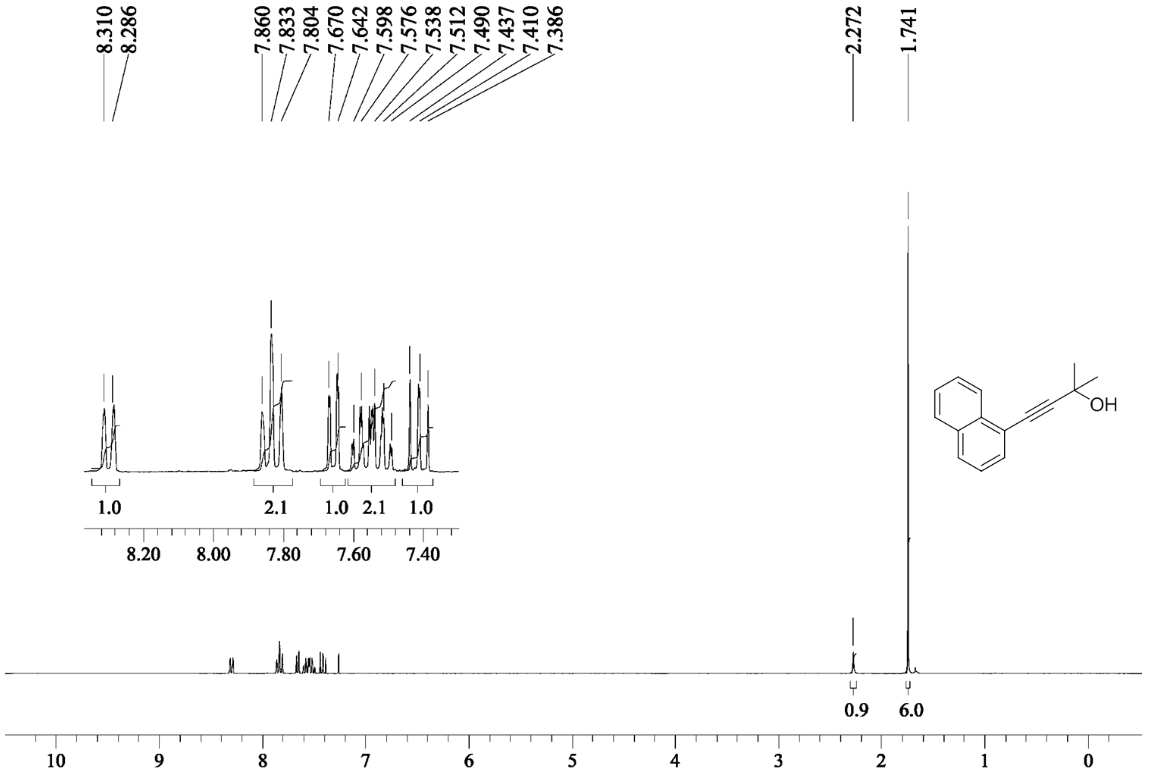

^{13}C NMR ($CDCl_3$, 75 MHz)

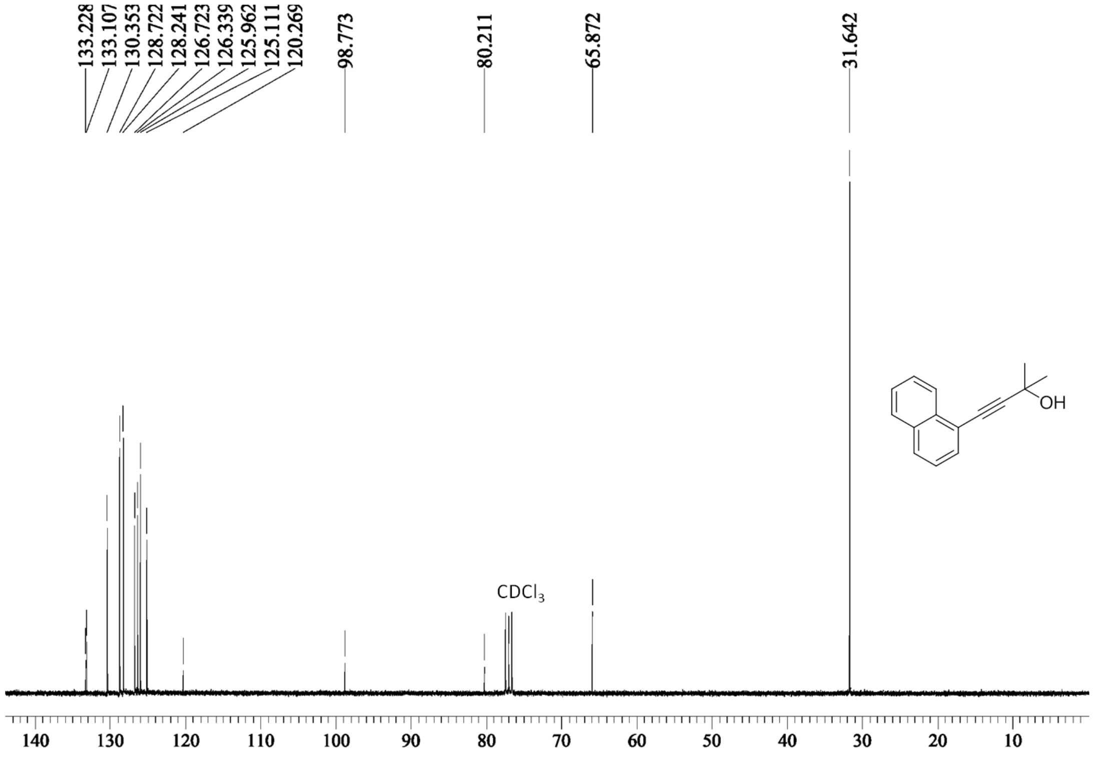

2-Methyl-4-(3-chlorophenyl)-3-butyn-2-ol (2k)

^{1}H NMR ($CDCl_3$, 300 MHz)

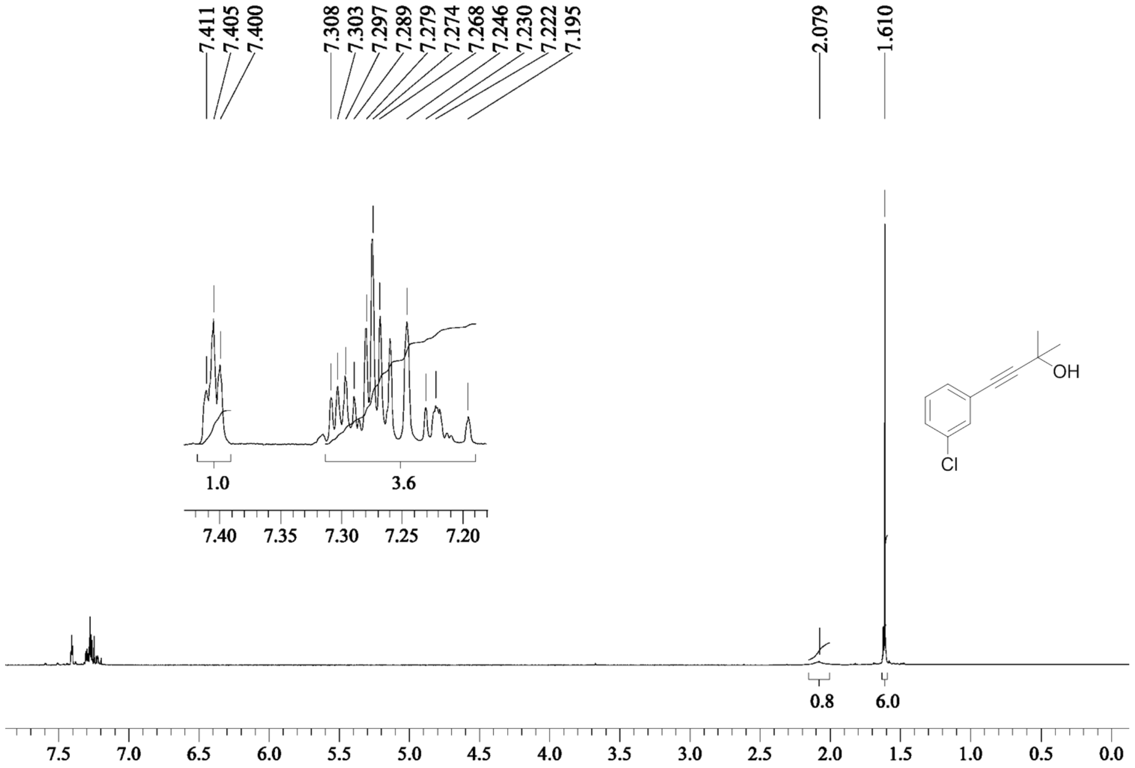

^{13}C NMR ($CDCl_3$, 75 MHz)

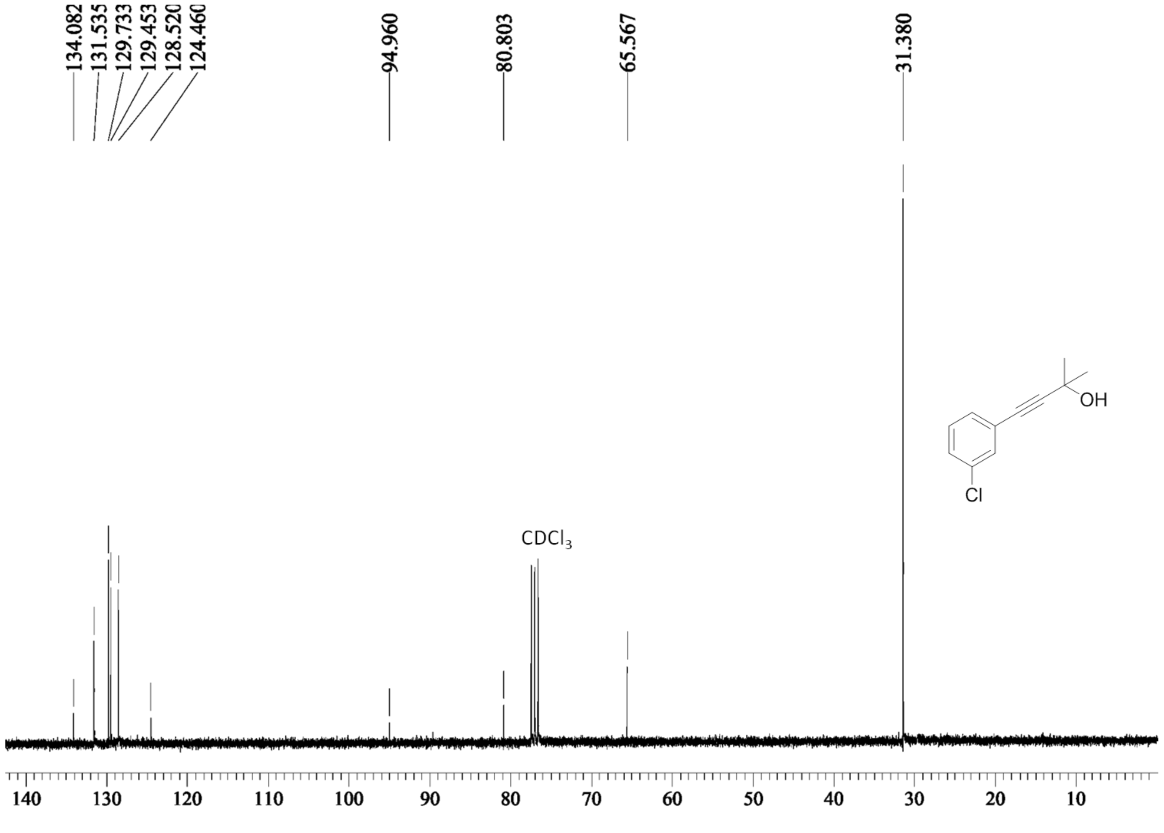

2-Methyl-4-(4-Fluorophenyl)-3-butyn-2-ol (2l)

^{1}H NMR ($CDCl_3$, 300 MHz)

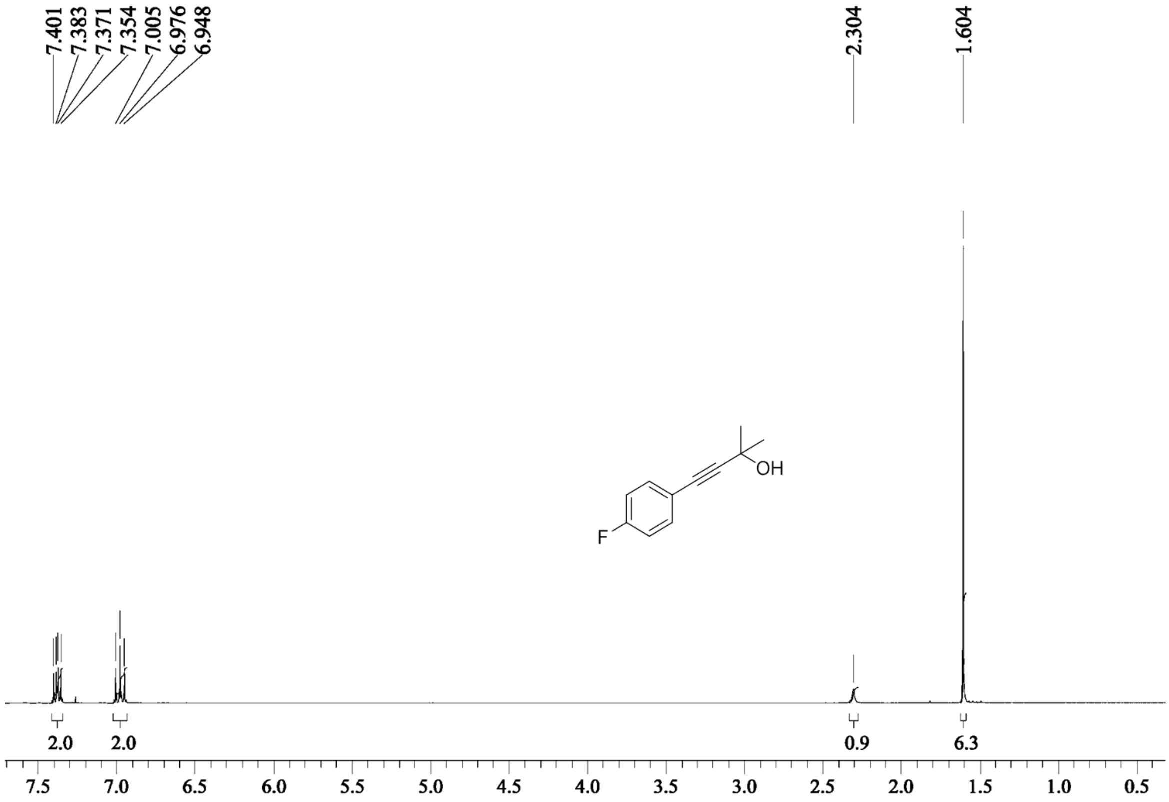

^{13}C NMR ($CDCl_3$, 75 MHz)

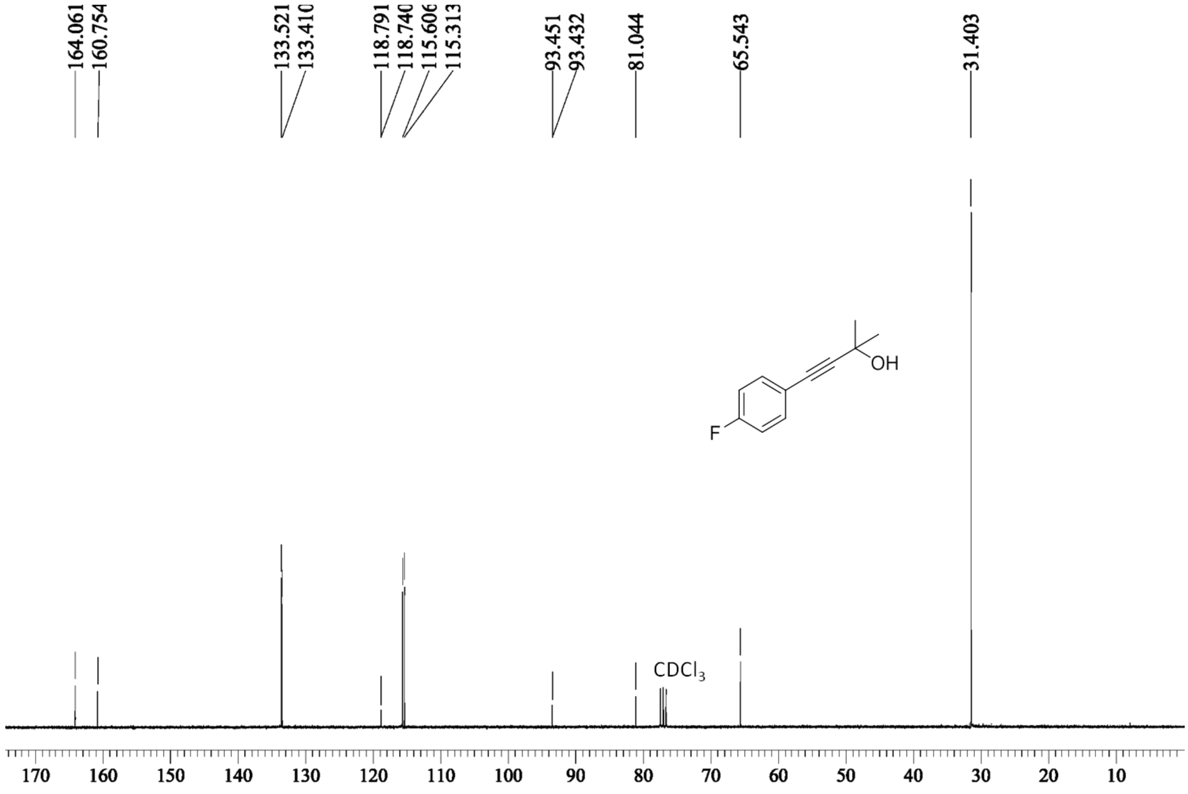

2-Methyl-4-(4-(trifluoromethyl)phenyl)-3-butyn-2-ol (2m)

^{1}H NMR ($CDCl_3$, 300 MHz)

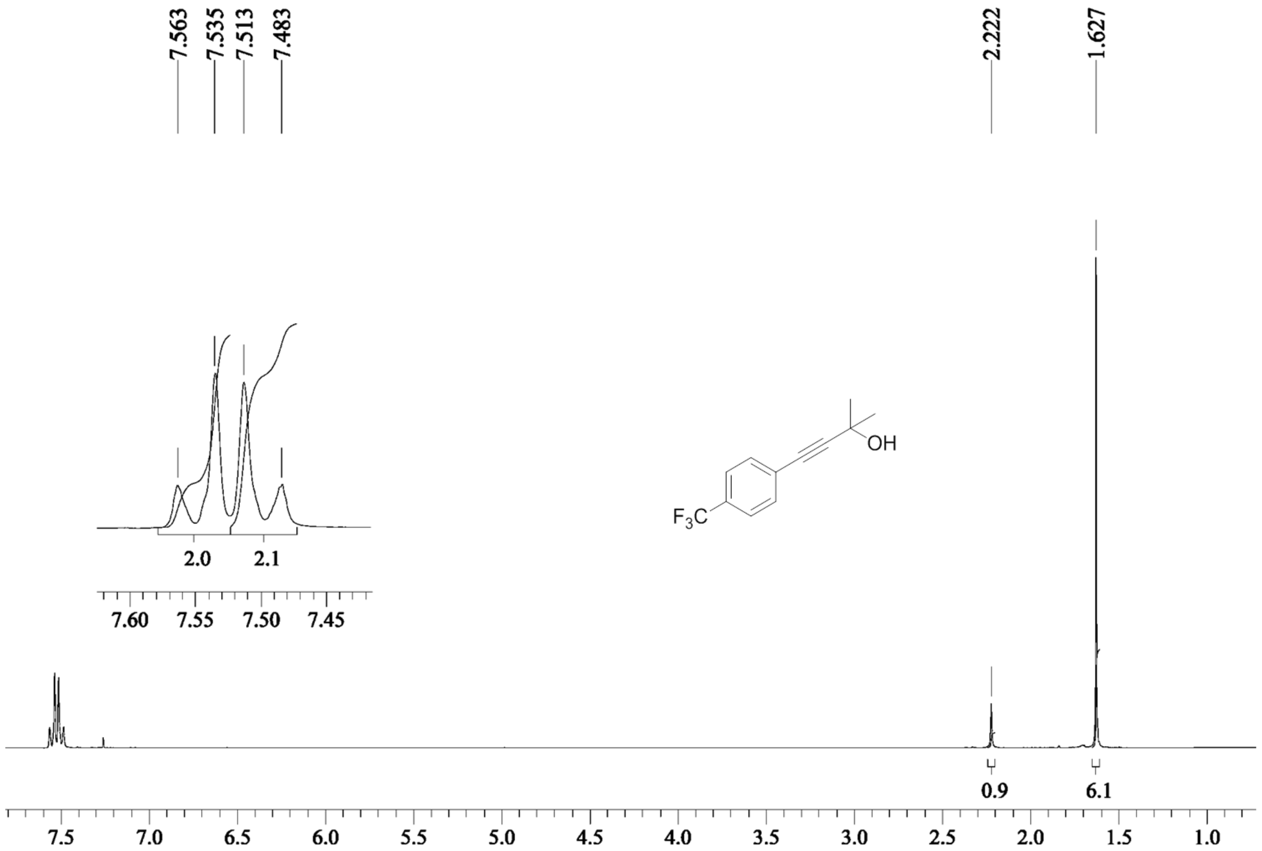

^{13}C NMR ($CDCl_3$, 75 MHz)

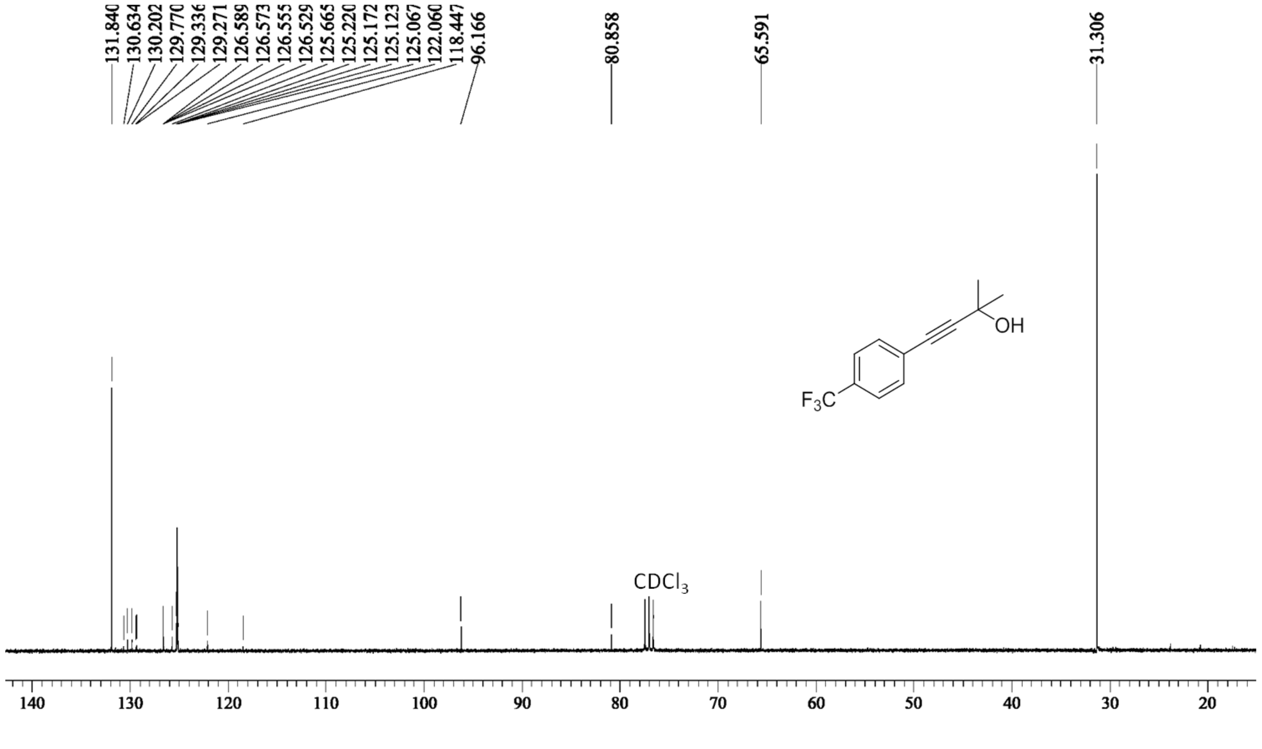

2-Methyl-4-(3-pyridinyl)-3-butyn-2-ol (2n)

^{1}H NMR ($CDCl_3$, 300 MHz)

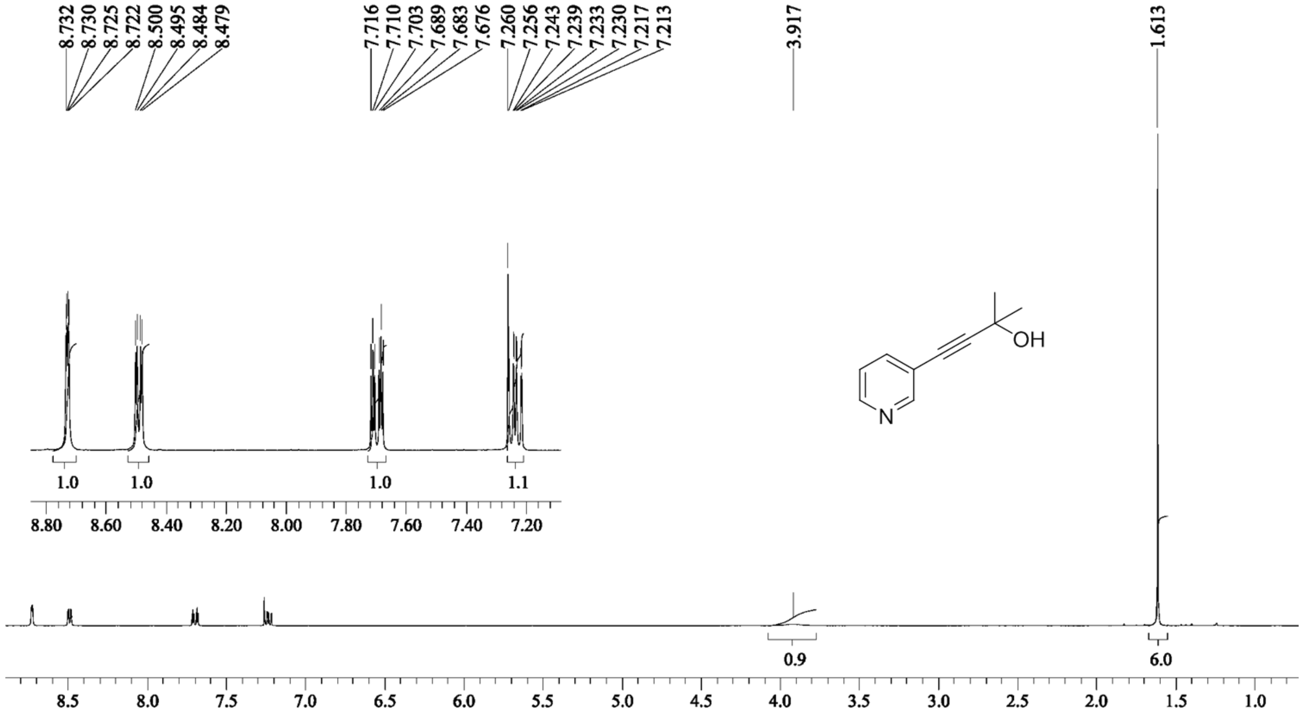

^{13}C NMR ($CDCl_3$, 75 MHz)

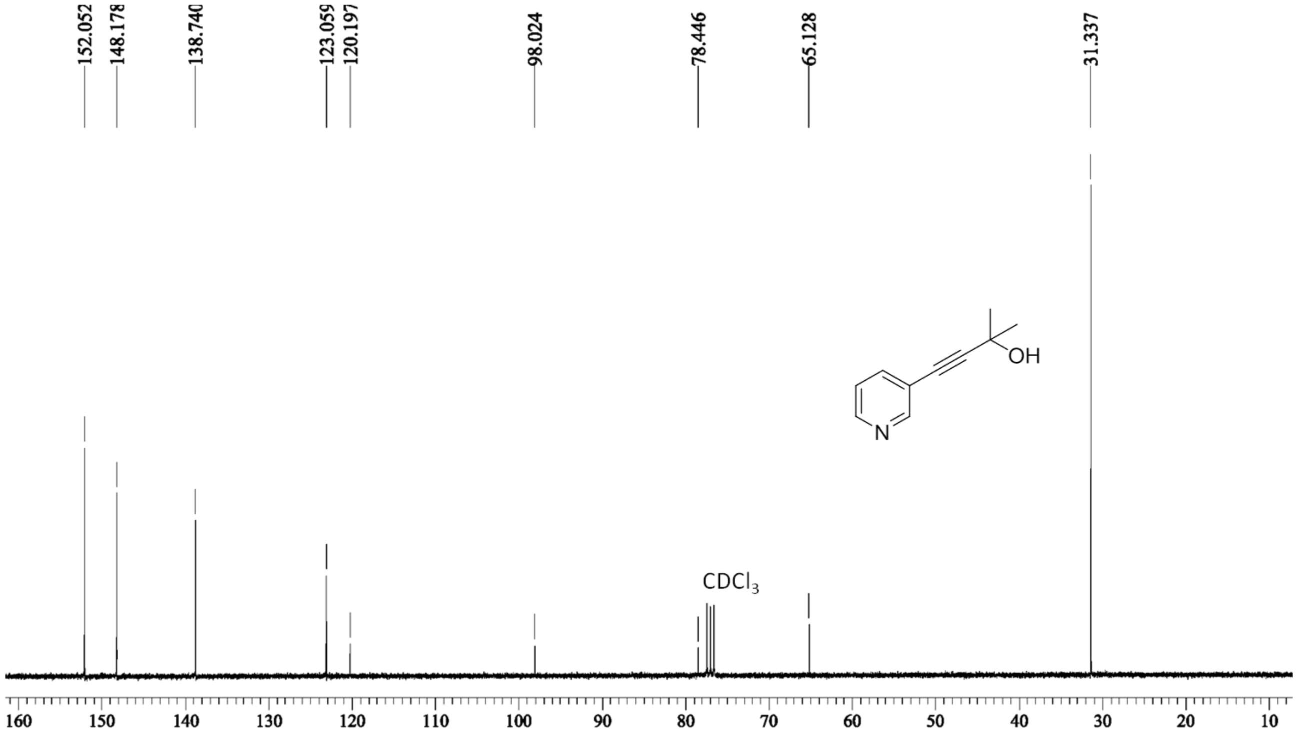

2-Methyl-4-(3-nitrophenyl)-3-butyn-2-ol (2o)

^{1}H NMR ($CDCl_3$, 300 MHz)

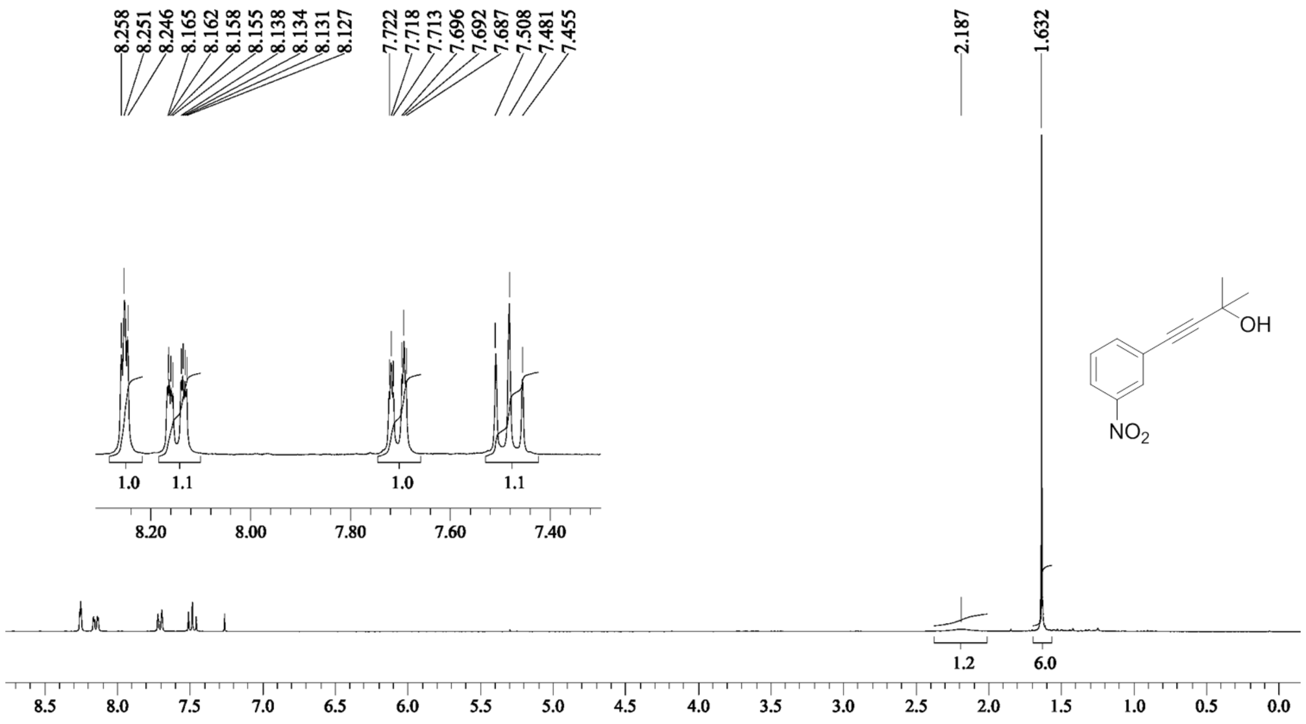

^{13}C NMR ($CDCl_3$, 75 MHz)

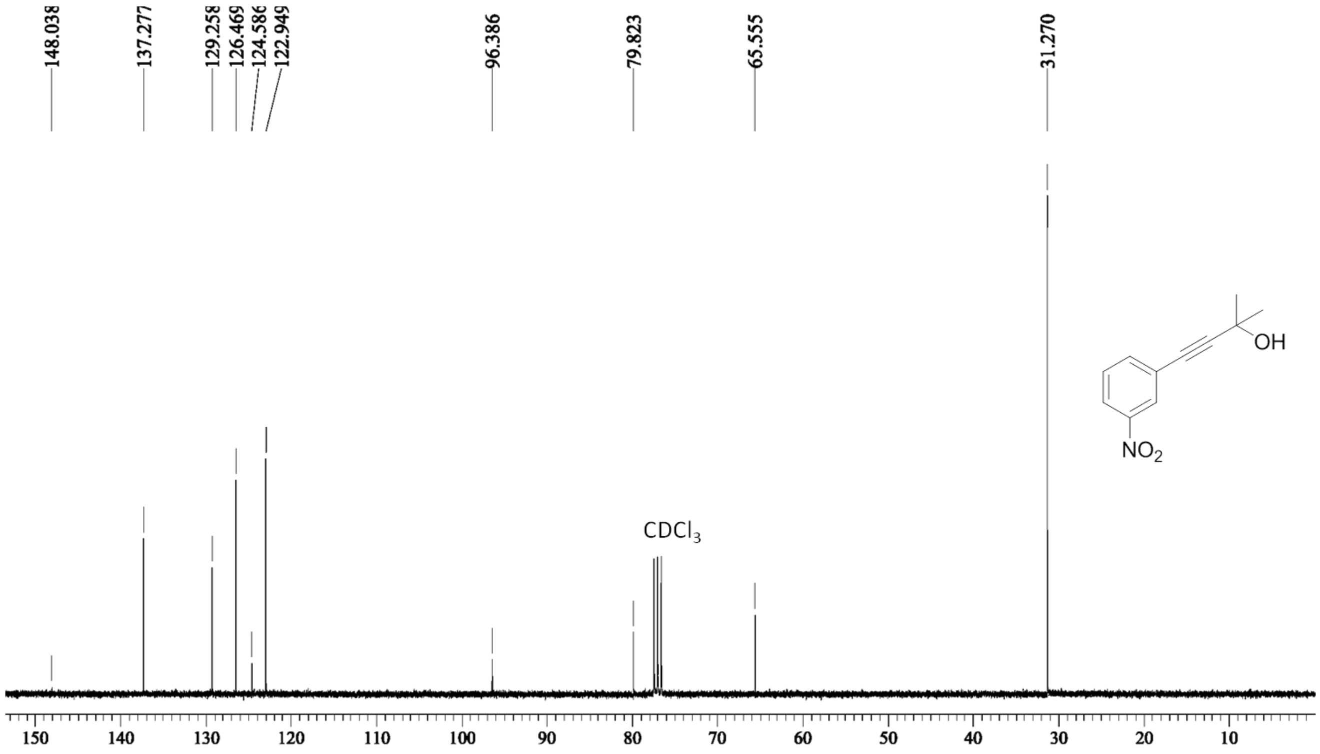

Methyl 4-(3-hydroxy-3-methylbut-1-ynyl)benzoate (2p)

^{1}H NMR ($CDCl_3$, 300 MHz)

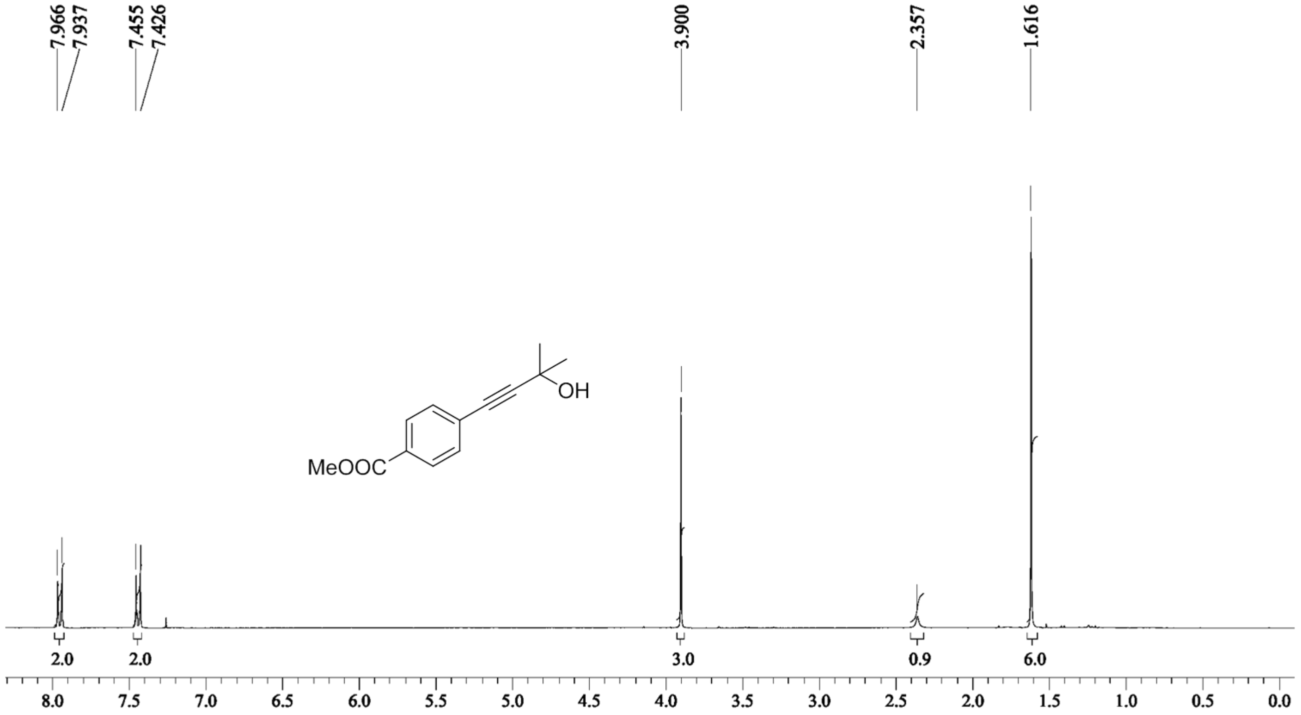

^{13}C NMR ($CDCl_3$, 75 MHz)

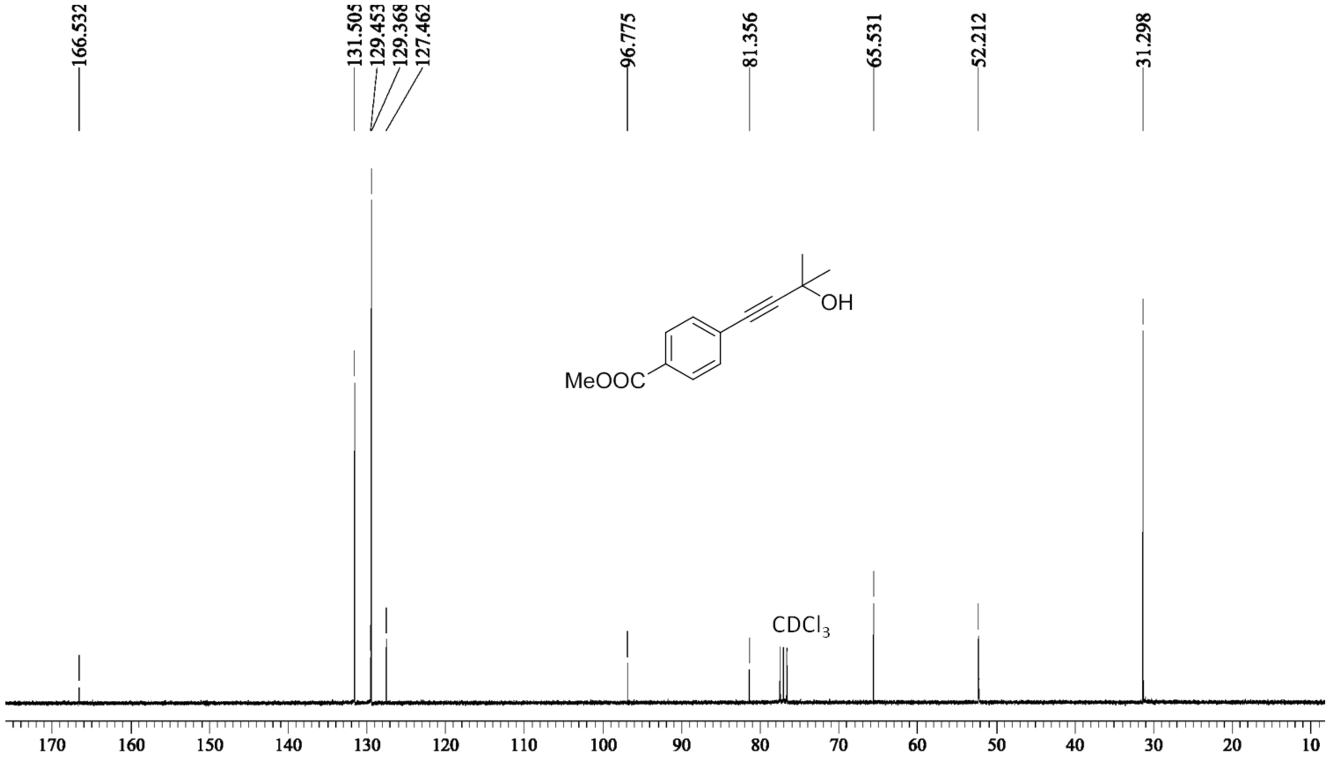

2-Methyl-4-(4'-cyanophenyl)-3-butyn-2-ol (2q)

^{1}H NMR ($CDCl_3$, 300 MHz)

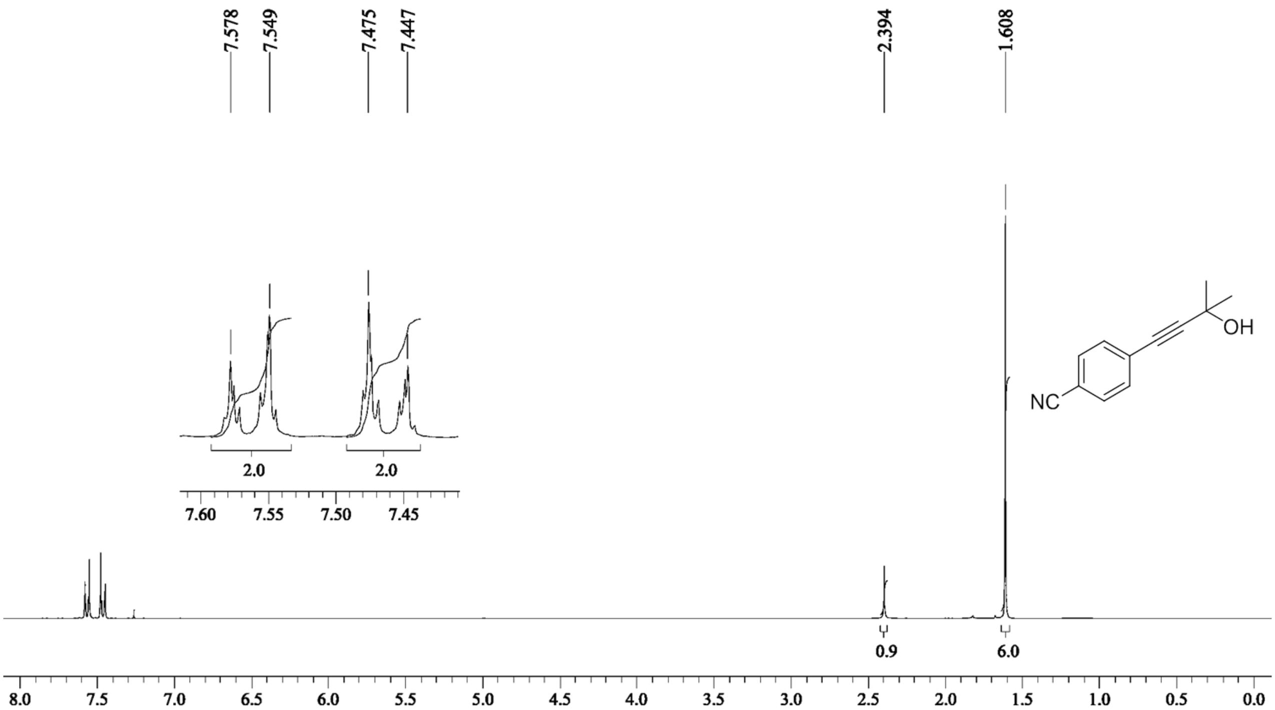

^{13}C NMR ($CDCl_3$, 75 MHz)

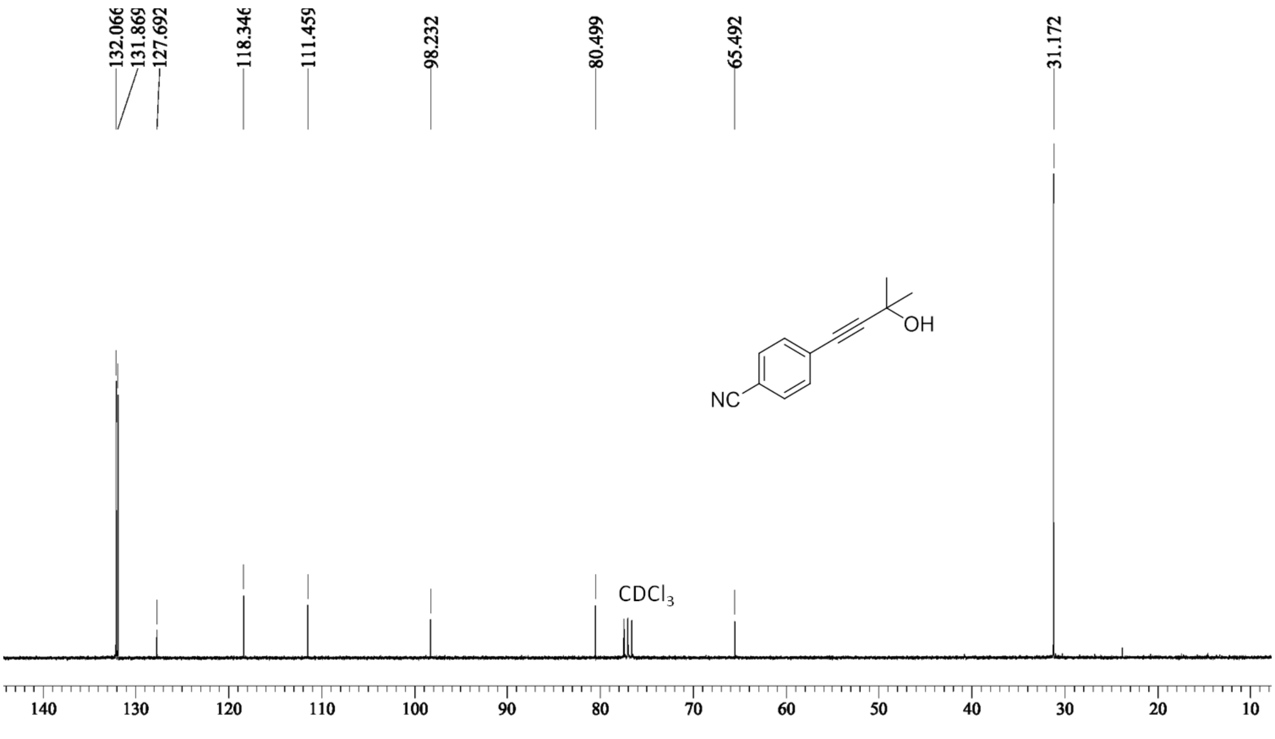

Supplement: File 1 — Description of all procedures and characterization data of all new compounds. [file Beilstein_J_Org_Chem-10-384-s001.pdf]
